# Enhancing ALS progression tracking with semi-supervised ALSFRS-R scores estimated from ambient home health monitoring

**DOI:** 10.3389/fdgth.2025.1657749

**Published:** 2025-09-26

**Authors:** Noah Marchal, William E. Janes, Sheila Marushak, Mihail Popescu, Xing Song

**Affiliations:** ^1^ Institute for Data Science and Informatics, University of Missouri, Columbia, MO, United States; ^2^ School of Medicine, Department of Biomedical Informatics, Biostatistics, and Medical Epidemiology, University of Missouri, Columbia, MO, United States; ^3^ Department of Occupational Therapy, University of Missouri, Columbia, MO, United States

**Keywords:** amyotrophic lateral sclerosis, disease progression, functional status, patient monitoring, personalized medicine, remote sensing technology, semi-supervised machine learning

## Abstract

**Introduction:**

Clinical monitoring of functional decline in amyotrophic lateral sclerosis (ALS) relies on periodic assessments, which may miss critical changes that occur between visits when timely interventions are most beneficial.

**Methods:**

To address this gap, semi-supervised regression models with pseudo-labeling were developed; these models estimated rates of decline by targeting Revised Amyotrophic Lateral Sclerosis Functional Rating Scale (ALSFRS-R) trajectories with continuous in-home sensor data from a three-patient ALS case series. Three model paradigms were compared (individual batch learning and cohort-level batch vs. incremental fine-tuned transfer learning) across linear slope, cubic polynomial, and ensembled self-attention pseudo-label interpolations.

**Results:**

Results showed cohort-level homogeneity across functional domains. For ALSFRS-R subscales, transfer learning reduced the prediction error in 28 of 34 contrasts [mean root mean square error (RMSE) = 0.20 (0.14–0.25)]. However, for composite ALSFRS-R scores, individual batch learning was optimal for two of three participants [mean RMSE = 3.15 (2.24–4.05)]. Self-attention interpolation best captured non-linear progression, providing the lowest subscale-level error [mean RMSE = 0.19 (0.15–0.23)], and outperformed linear and cubic interpolations in 21 of 34 contrasts. Conversely, linear interpolation produced more accurate composite predictions [mean RMSE = 3.13 (2.30–3.95)]. Distinct homogeneity-heterogeneity profiles were identified across domains, with respiratory and speech functions showing patient-specific progression patterns that improved with personalized incremental fine-tuning, while swallowing and dressing functions followed cohort-level trends suited for batch transfer modeling.

**Discussion:**

These findings indicate that dynamically matching learning and pseudo-labeling techniques to functional domain-specific homogeneity-heterogeneity profiles enhances predictive accuracy in tracking ALS progression. As an exploratory pilot, these results reflect case-level observations rather than population-wide effects. Integrating adaptive model selection into sensor platforms may enable timely interventions as a method for scalable deployment in future multi-center studies.

## 1 Introduction

Amyotrophic lateral sclerosis (ALS) is a neurodegenerative disease affecting the motor neuron system, with patients experiencing significant difficulties performing across a range of functions, resulting in a reduced ability for self-care. Decline in function is measured regularly at provider visits using clinical instruments like the Revised Amyotrophic Lateral Sclerosis Functional Rating Scale (ALSFRS-R) (1). However, acute functional decline may go undetected by clinicians until the next follow-up due to the long duration between office visits. Sensor monitoring, which has been shown to be effective in supporting care for older adults living independently, offers a possible solution for tracking functional changes related to disease progression in those living with ALS. Sensor measurements may serve as predictive features to target instrument scales over interim periods between clinic visits, thereby increasing the fidelity of functional measures to aid clinicians in making better, more informed care strategies to guide interventions. In this study, we trained and evaluated three semi-supervised learning models (participant-level batch, cohort-level transfer with batch, and incremental fine-tuning) across three pseudo-label techniques (linear, cubic, and self-attention interpolation) to predict ALSFRS-R scale trajectories from in-home sensor health features using root mean square error (RMSE) and Pearson’s correlation (r) as primary metrics of model accuracy and fit.

### 1.1 Sensor monitoring of ALS progression

Sensor-based health monitoring has been shown to improve clinical outcomes in older adult independent living residents through early illness detection, enabling them to maintain their independence longer (2). Physical deficits in older adults may mirror the functional declines observed in ALS, with community-dwelling older adults experiencing a stable physical function until a steep decline 1–3 years before death (3). Additionally, age-related frailty may involve motor unit loss (denervation) similar to ALS, which contributes to muscle wasting and could further exacerbate ALS progression in older patients (4). This evidence indicates that remote sensor monitoring technologies effective for improving care in elder populations may identify digital biomarkers for tracking ALS disease progression. Recent research has found that combining wearable sensor data with self-reported clinic assessments and environmental metrics improves predictive models targeting ALSFRS-R scales (5). Similarly, work evaluating wearable accelerometer, ECG, and digital speech sensors for tracking ALS has shown that changes in physical activity, heart rate, and speech features correlate with a decline in ALSFRS-R scales (6). More frequent, remote sensor-based tracking of changes in ALSFRS-R scales would enable clinicians to better target interventions and detect acute events, such as falls or medication changes, between clinic visits.

### 1.2 Clinical use of ALSFRS-R scales

ALS disease progression rates vary between patients due to a number of clinical factors including baseline functional status, disease stage at diagnosis and diagnostic delay, co-occurrence of frontotemporal dementia, gender, age and site at onset, particularly respiratory-onset, and a number of genetic and environmental factors (7–9). Disease progression also varies across functional domains within ALS patients, following non-linear rates of decline in specific areas (10). ALS progression is tracked longitudinally using the ALSFRS-R instrument as a qualitative, subjective self-reported measure of performance in functional tasks. ALSFRS-R scales are collected during clinic visits to determine the amount of change in bulbar, fine motor, gross motor, and respiratory functional domains over time. Scales are rated between 0 and 4, with 0 indicating dependence and 4 indicating no difficulty. The composite score and linear slope serve as primary metrics of functional change and decline progression and for measuring intervention effects within individuals or across treatment groups in clinical trials, with more frequent assessment improving slope estimation (11, 12). Due to the multi-dimensional aspect of the aggregate ALSFRS-R composite score, it has been suggested to use the component scales independently for measuring treatment outcomes (13). As such, there is not a one-size-fits-all approach for monitoring progression, as decline varies non-linearly among patients, and individualized clinical models are needed for tracking across ALSFRS-R functional domains.

## 2 Materials and methods

### 2.1 Parent study

Participants were recruited for a single-site, single-cohort prospective study overseen by the MU Institutional Review Board through the MU Health ALS Clinic, investigating continuous, in-home sensor monitoring for tracking between-visit functional decline (14). The in-home sensor monitoring systems, licensed by the University of Missouri to Foresite Healthcare, LLC, are composed of three modalities for continuous contactless data collection: bed mattress hydraulic transducers for recording ballistocardiogram (BCG)-derived respiration, pulse, and sleep restlessness measures (15, 16); privacy-preserving thermal depth sensors (17, 18), which detect falls and collect walking speed, stride time, and stride length measurements, although gait data were excluded due to wheelchair use; and passive infrared (PIR) motion sensors that provide room activity counts.

Inclusion criteria required an ALS diagnosis, residence within 100 miles of the clinic, and either a home caregiver or a Montreal Cognitive Assessment (MoCA, 8.1 Blind Version) cutoff score of ≥19 out of 22, corresponding to the standard cutoff of ≥26/30 on the full MoCA. ALSFRS-R scores were collected monthly by telephone and quarterly as pre-clinic assessments. After accounting for length of enrollment, data from three participants were of sufficient duration (at least 6 months) for case series modeling, as shown in Table 1. All three participants were non-Hispanic, white, male, Medicare recipients, who left the study due to death. At the time of enrollment, their ALSFRS-R composite scores ranged from 30 to 35, indicating moderate functional impairment. P1 presented with lateral onset (extremity weakness and spasms), P2 with cervical-bulbar and limb weakness, and P3 with bulbar speech changes accompanied by lateral weakness. Diagnosis was determined in the clinic using the revised El Escorial and Awaji diagnostic criteria workflow for ALS (19) and confirmed for study participation by the presence of SNOMED CT codes for ALS (86044005, 142653015, 62293019) and ICD10 (G12.21) in the patient’s medical chart. With regard to disease progression timelines, the interval from diagnosis to enrollment varied among participants (from 24 to 623 days), reflecting the heterogeneous nature of the disease. The use of assistive devices and non-invasive ventilation (NIV) also differed, with P1 requiring ankle-foot orthosis (AFO), walker/wheelchair, and eventually a powered wheelchair over a prolonged period (504–763 days from diagnosis). P2 and P3 were diagnosed at a later ALS stage and had more rapid progressions with shorter intervals to assistive device use (P2: powered wheelchair at 247 days, P3: walker or wheelchair at 76 days) and death. NIV initiation ranged from 14 to 1,127 days post-diagnosis.

**Table 1:** Participant enrollment and dataset characteristics.

Characteristic	P1	P2	P3
ALS profile			
Age at enrollment (years)	62	55	45
Onset site	Lateral	Bulbar	Bulbar
Initial study ALSFRS-R composite score	35	33	30
Clinical timeline (days from Dx)			
Enrollment	623	24	56
Initial study ALSFRS-R	623	22	50
Assistive device			
AFO	504	—	—
Walker or wheelchair	714	—	76
Powered wheelchair	763	247	—
NIV	1127	234	14
Death	1222	260	275
Dataset length			
ALSFRS-R assessments (n)	15	8	8
Enrollment length (days)	599	236	219
Training dataset (days)	389	128	156
Test dataset (days)	98	33	40

**Figure 1:**
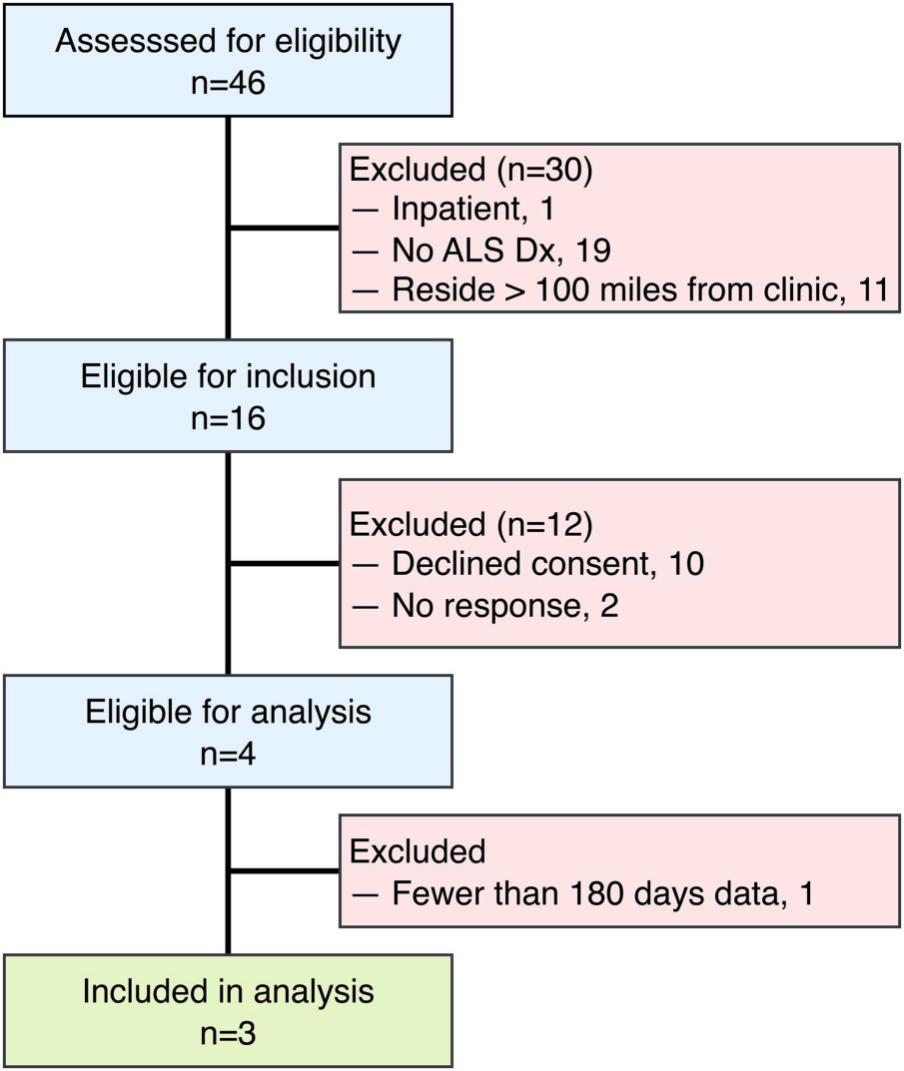
Flowchart depicting the participant inclusion and exclusion process for the study.

Given the small sample, these findings should be interpreted as case-level observations with limited generalizability, rather than being extrapolated to population-level ALS progression. The limited sample size (n=3) of this study reflects both the rarity and rapid progression of ALS, as well as practical limitations specific to remote sensor research within this patient population. Participants were screened for eligibility during the study recruitment period, as outlined in Figure 1. Individuals were excluded for inpatient status, lack of ALS diagnosis, and residing further than 100 miles from the MU ALS Clinic. Additional exclusions occurred due to non-response to recruitment or by declining consent. The inclusion criterion requiring residence within 100 miles of the clinic was selected due to logistical considerations for sensor installation and maintenance rather than intent to restrict sampling. Only those having at least 6 months of monitoring data were included in the analytic sample. Of the 16 individuals who passed eligibility criteria, 10 declined participation due to privacy or structural concerns about sensor installation, 2 did not respond to recruiting materials, and only 3 of the 4 enrolled completed at least 6 months of data collection sufficient for analysis. Despite the small cohort size, participants contributed extensive clinical and sensor-based data, offering within-individual longitudinal detail that is characteristic of ALS observational studies. A larger, multi-site trial is being planned to address scalability and generalizability in future research.

### 2.2 Estimating between-visit change in ALSFRS-R scales

**Figure 2:**
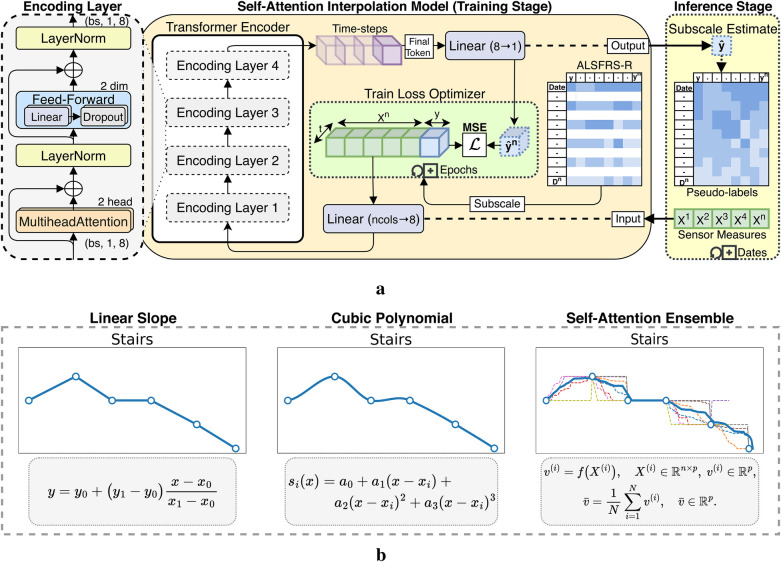
Interpolation techniques applied to ALSFRS-R subscores for estimating sensor feature pseudo-labels. **(a)** Self-attention interpolation transformer architecture. **(b)** Comparison of interpolation effects.

**Figure 3:**
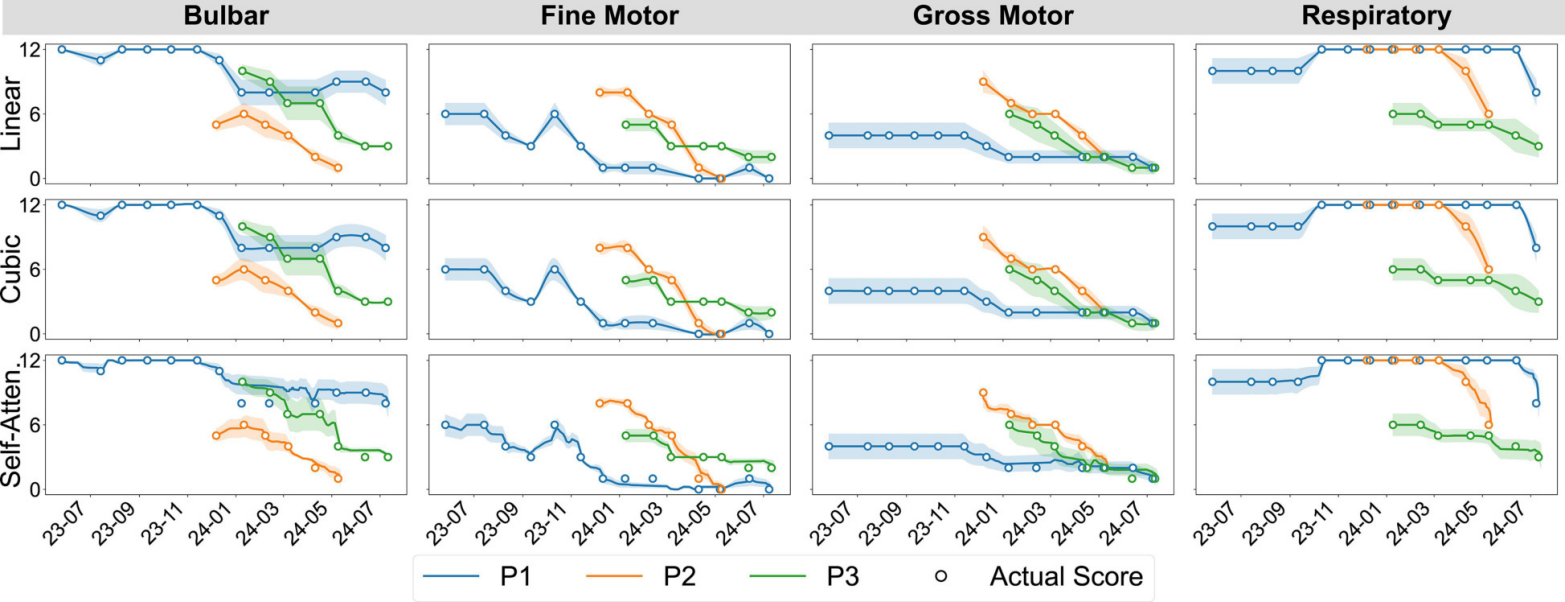
Participant-aggregated ALSFRS-R subscores by the functional domain and interpolation technique.

ALSFRS-R scales were aligned at matching frequency to daily aggregated sensor measurements using pseudo-labels for semi-supervised regression, extending prior work evaluating between-visit interpolation (20). We incorporated a transformer encoder architecture for self-attention interpolation, which we compared to polynomial functions, as illustrated in Figure 2a. Linear 1D piecewise interpolation served as a baseline method, consistent with the clinical methodology for tracking ALS progression. Non-linear cubic spline interpolations were applied to evaluate more gradual rates of decline. The transformer encoder mapped date-indexed sensor vectors with known ALSFRS-R scores to estimate the amount of change occurring between collection points, with the architecture intentionally kept shallow to provide continuous values rather than predicting crisp labels with a deeper network (21). To further smooth the estimations, self-attention interpolation was applied to each sensor feature algorithm table and then ensembled by averaging, as shown by the dashed plots in Figure 2b. The resulting interpolated slopes over time for each pseudo-labeling technique, which are summated by functional area in Figure 3, demonstrate varying rates of decline unique to each participant.

### 2.3 Semi-supervised learning of ALSFRS-R scales

Three learning approaches for training semi-supervised regression models were compared: batch models fit on sequential participant-level data (individual) and cohort-level (transfer) models initialized on randomized observations and fine-tuned on individual-level data using batch or incremental learning (22). Participant-subscales exhibiting zero or near-zero variance in training samples were not modeled with individual batch learning.

#### 2.3.1 Data preprocessing and feature engineering

**Figure 4:**
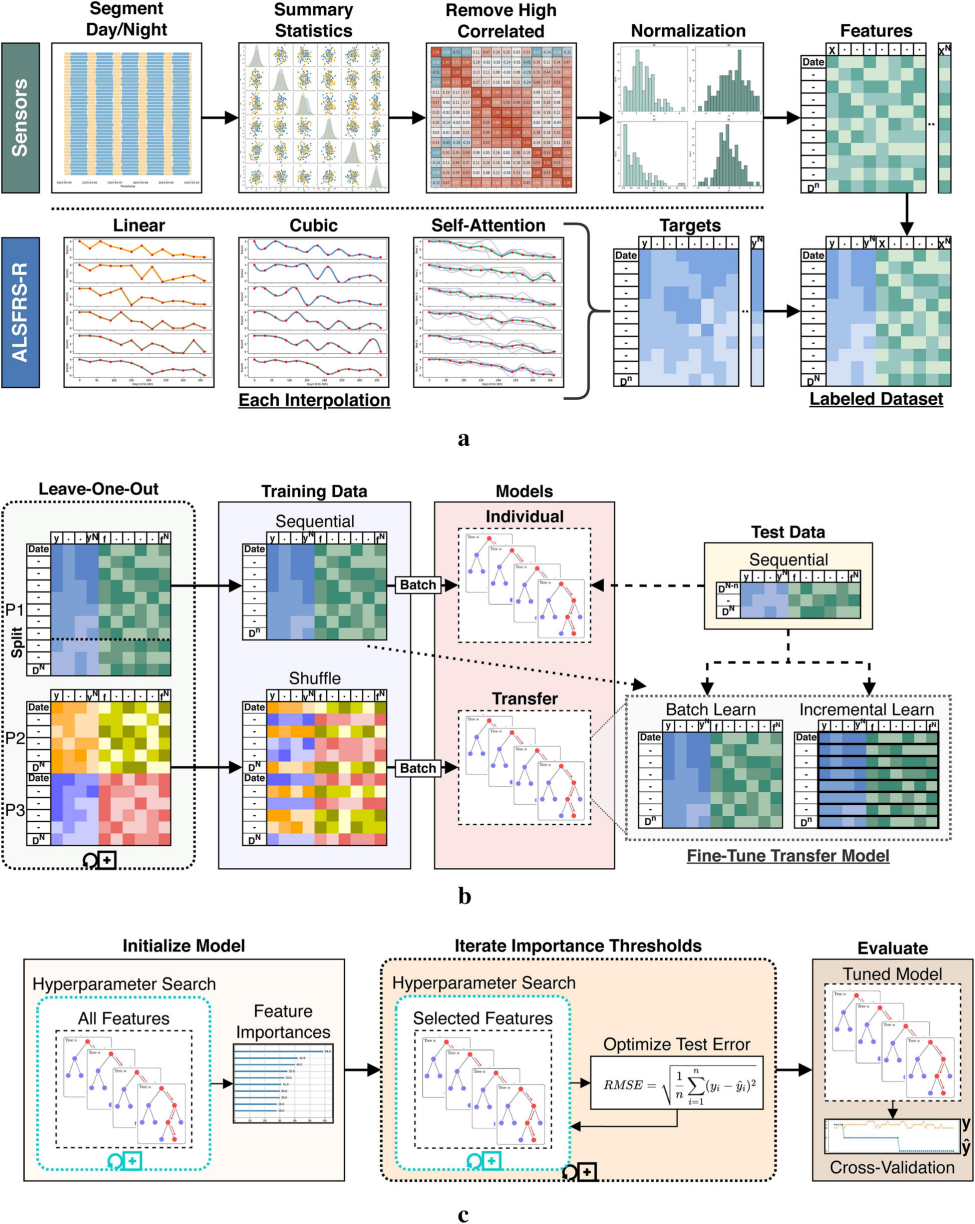
Data processing and model fitting pipeline. **(a)** Sensor and ALSFRS-R preprocessing steps. **(b)** Dataset segmentation for individual batch and cohort transfer learning models. **(c)** Iterative model tuning and evaluation stages.

High-frequency sensor data were preprocessed using the pipeline described in Figure 4a, beginning with segmentation of the time-indexed features into day and night periods. Summary statistics were calculated over each feature channel and period for count, minimum, maximum, mean, median, mode, variance, range, skew, kurtosis, quantiles, interquartile range (IQR), coefficient of variation (CV), and entropy to better capture temporal patterns by time-of-day. The selected features were chosen based on established use of time-series features in clinical prediction and prior wearable sensor research, where summary statistics have been shown to effectively capture both overall trends and subtle changes in physiological and behavioral signals relevant to disease progression (23, 24). As a case series pilot for continuous in-home sensor monitoring in ALS, we first prioritized conventional summary statistics for baseline modeling and then relied on native feature selection to identify relevant ALSFRS-R predictors. Highly collinear features were then removed, and the resulting set was normalized feature-wise using a minimum–maximum scaling. ALSFRS-R scores were interpolated with each pseudo-labeling technique, resulting in three continuous target trajectories per participant per scale. Finally, the normalized features and interpolated labels were joined by date to produce the pseudo-labeled datasets.

#### 2.3.2 Individual batch and cohort transfer learning

Individual- and cohort-level models were trained using a leave-one-participant-out strategy, as shown in Figure 4b. For each experiment fold, a single participant (P1, P2, or P3) was withheld as the target subject, while data from the remaining participants formed the source cohort for transfer model training. Each holdout participant’s dataset was split sequentially by 80%/20%. The earliest 80% of observations were used for both training the individual batch model and fine-tuning the cohort transfer model, while the later 20% were set aside as an unseen test set for model evaluation. This temporal partitioning was chosen to reflect the use case of predicting future ALSFRS-R scores based on prior sensor data and prevent data leakage. To ensure fair comparison between models, training and test splits were defined proportionally to each individual’s data, not by group. Individual models were trained using batch learning on the holdout participant’s sequential training data. Transfer learning was conducted by first training a cohort model on the leave-in participants’ data using batch learning with shuffled samples to capture generalizable patterns across individuals. This cross-individual split simulated the scenario of applying knowledge learned from a group to a new, unseen individual. The resulting model parameters (including learned weights and optimizer state) were serialized by pickling and storing the model object. For each participant fold, the initialized transfer model was then reloaded and further adapted using the sequential training data from the holdout participant. This adaptation was done using batch and incremental learning routines, comparatively. Transfer batch fine-tuned models were updated with a single pass through the entire training set of the holdout participant. Conversely, transfer incremental models were trained iteratively by predicting the current outcome label and then fitting the new observation to simulate between-visit model adaptation as additional data become available. All transferred model components were included in the fine-tuning step. Hyperparameters for these fine-tuned models (e.g., learning rate, batch size, optimizer type) were held constant from the initialized model during subsequent fine-tuning and were selected using cross-validation. As shown in Table 1, participants differed in their dataset length and number of ALSFRS-R assessments collected. To prevent bias, all model evaluations were performed within-participant, and prediction errors and outcome correlations were computed only on the holdout subject’s test data for each fold. We did not aggregate or compare metrics across participants. This approach was chosen to focus on the model’s ability to estimate patient-specific ALS disease progression and to infer homogeneous–heterogeneous profiles across ALSFRS-R scales.

#### 2.3.3 Iterative hyperparameter tuning and feature selection

To evaluate the effects of label interpolation and transfer learning strategies within a consistent modeling framework, rather than to benchmark across diverse machine learning algorithms, a single learner experiment design was chosen to evaluate pseudo-labeling interpolations and transfer model adaptations. We employed an iterative screener-learner approach combining hyperparameter optimization with feature selection, illustrated in Figure 4c, using the XGBoost algorithm (25). Models were initialized on the full set of summary features and hyperparameter tuned using *RandomizedSearchCV* in the *Scikit-Learn* Python package. Following initialization, feature importance scores were extracted using feature weight frequency internal to XGBoost. Importance scores were then binned through iterative precision-rounding, beginning at six decimal places and consecutively increasing precision levels to a maximum of 200 features to avoid overfitting. For each feature subset identified, a new model was fit using the selected features and tuned hyperparameters, as specified in Table 2. The root mean square error (RMSE) was calculated and compared across all iterations, with the model configuration yielding the lowest test RMSE selected as the optimized model. The best-performing transfer model was then fine-tuned on the holdout participant’s training set using the trained booster as a checkpoint to resume learning.

**Table 2:** Hyperparameter search spaces for tuning the XGBoost classifier algorithm.

Hyperparameter	Description	Search space
eta (learning rate)	Learning rate to scale the contribution of each tree	[0.001, 0.01, 0.1, 0.3, 0.5]
n_estimators	Number of boosting rounds (trees) to build	[32, 64, 128, 192, 256, 384, 512]
gamma	Minimum loss reduction required to perform a split	[0, 0.25, 0.5, 1]
max_depth	Maximum depth of trees to prevent overfitting	[2, 3, 4, 6, 8, 10, 12, 16, 24]
min_child_weight	Minimum sum of instance weights needed in a child node	[0.5, 1, 3, 5, 7, 10]
subsample	Fraction of training data used for building each tree	[0.8, 0.9, 1.0]
colsample_bytree	Fraction of features randomly sampled for each tree	[0.6, 0.7, 0.8, 0.9]
lambda (reg_lambda)	L2 regularization to penalize large weights	[0.01, 0.1, 1, 5, 10, 50, 100]
alpha (reg_alpha)	L1 regularization to encourage sparsity in feature weight	[0, 0.001, 0.01, 0.1]

### 2.4 Model evaluation metrics and Taylor diagrams

Prediction performance metrics for the RMSE (prediction error), Pearson’s r (outcome correlation), and their confidence intervals (CIs) were calculated from modeled outcomes ($\hat{y}$) against the test values (y) that were held out from the model training data. Confidence intervals for the Pearson correlation were obtained by first applying Fisher’s z-transformation to stabilize the sampling distribution and computing the standard error, before constructing the 95% limits in z-space, and then converting these limits back to the correlation scale. If fewer than four points were available, the correlation interval was marked as missing. For RMSE confidence intervals, we used the non-parametric bootstrap method with 1,000 resamples of the paired true and predicted values, recalculated the RMSE for each resample using the standard deviation of true and predicted values along with their absolute correlation, and then took the 2.5% and 97.5% percentiles of the resulting RMSE distribution as the confidence bounds. Average summary metrics for ALSFRS-R subscales and the composite score were calculated by taking the mean value across participants, with the 95% confidence interval estimated using the standard error of the mean and the *t*-distribution. This provides an interval estimate that reflects the expected variability in case series cohort model performance. Comparisons between pseudo-labeling techniques and learning methods were made using Taylor diagrams to assess prediction accuracy, correlation, and variability at the cohort level by averaging outcomes across participants for each subscale. In these Taylor diagrams, the reference point on the X-axis represents the actual values (the estimated ALSFRS-R scale) plotted at coordinates $\left(\sigma_{\text{ref}},0\right)$, where $\sigma_{\text{ref}}$ is the standard deviation of the actual values, while the origin (0,0) represents a modeled prediction with SD = 0 and r=0 to the reference vector (26).

## 3 Results

**Figure 5:**
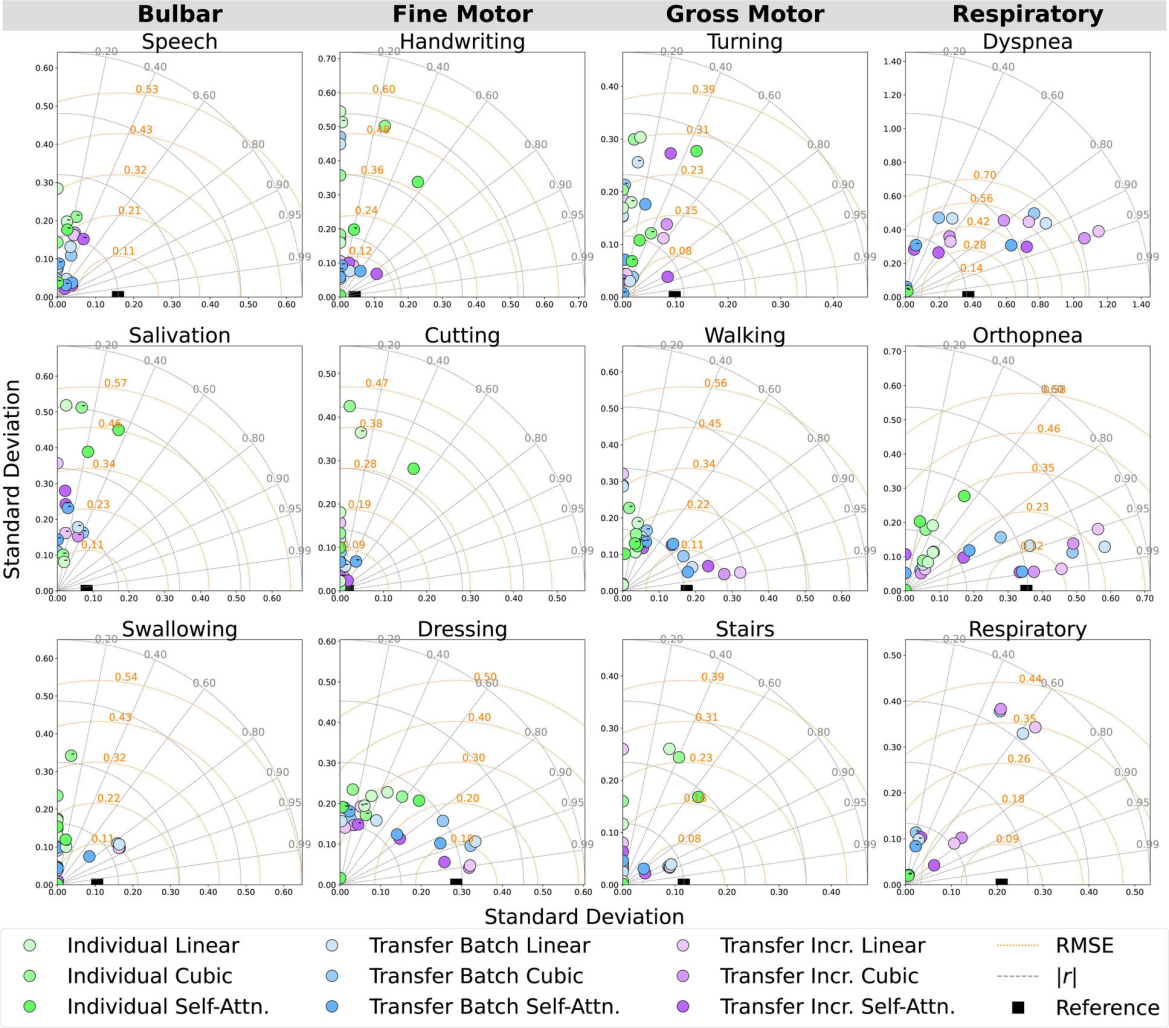
Participant-level Taylor diagrams depicting the mean RMSE, absolute correlation (|r|), and standard deviation of the predicted outcomes for each ALSFRS-R scale, annotated by negative (−) correlation.

**Figure 6:**
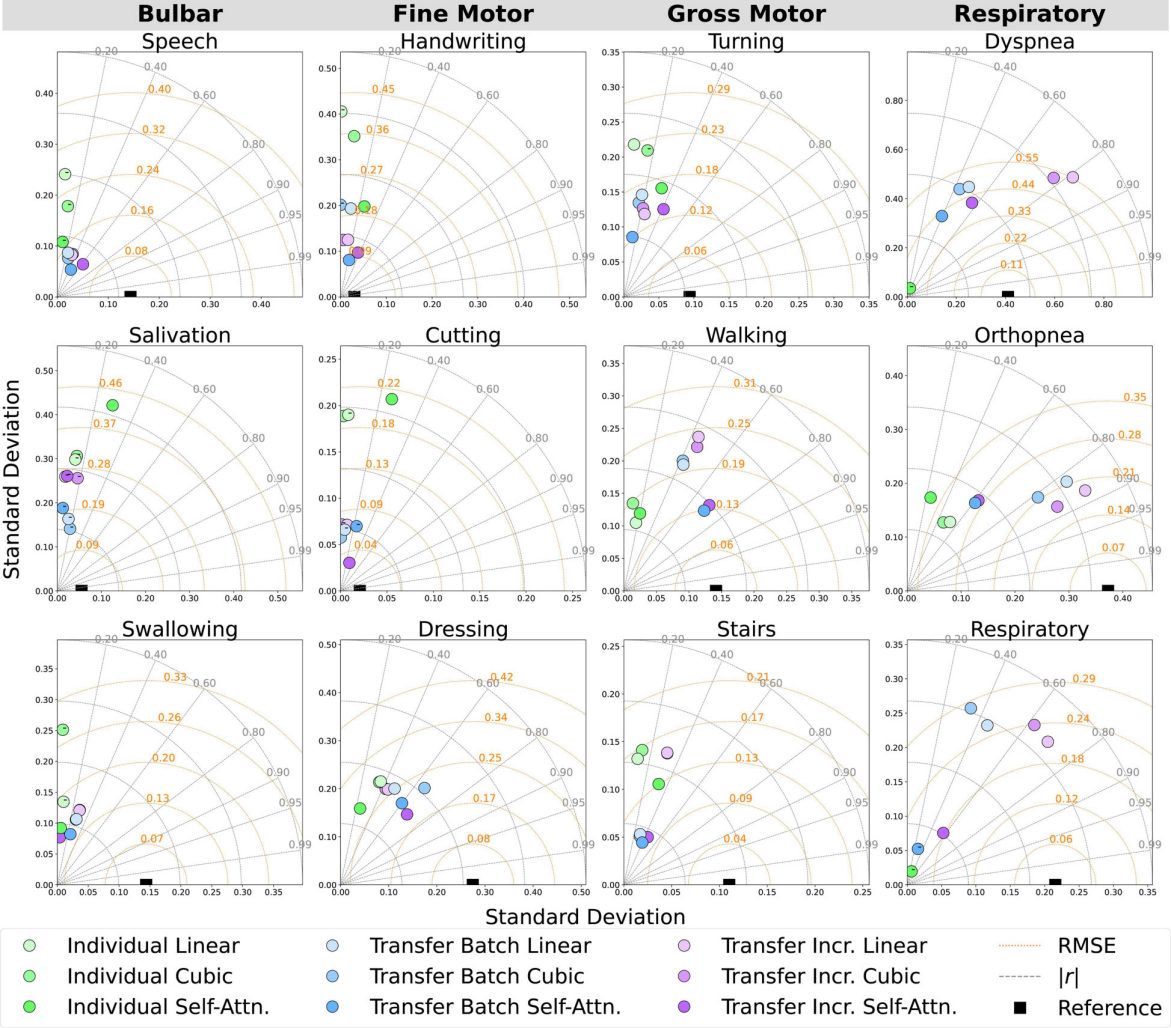
Cohort-averaged Taylor diagrams depicting the mean RMSE, absolute correlation (|r|), and standard deviation of the predicted outcomes for each ALSFRS-R scale, annotated by negative (−) correlation.

Models were evaluated across pseudo-labeling techniques (linear, cubic, self-attention interpolation) and learning methods (individual batch, transfer batch fine-tuned, transfer incremental fine-tuned) for predicting ALSFRS-R component and composite scales using in-home health sensor features; an asterisk (*) was used to mark significant improvements in these comparisons. Prediction errors and outcome correlations for ALSFRS-R subscales are illustrated by participant as Taylor diagrams in Figure 5 and cohort average in Figure 6. Low variances in collected scores, provided in Table 3, prevented the fitting of participant-subscale models for salivation in P1, speech in P2, dyspnea and respiratory for P1 and P2 for all interpolations, dyspnea and respiratory in P3 with linear and cubic interpolation, and cutting with self-attention interpolation. As P1’s salivation and respiratory scales had zero variance with static scores of 4, transfer models were not fit for these subscales.

**Table 3:** Observed variance in ALSFRS-R scales by participant.

Pt.	Bulbar			Fine motor			Gross motor			Respiratory		
	Speech	Salivation	Swallowing	Handwriting	Cutting	Dressing	Turning	Walking	Stairs	Dyspnea	Orthopnea	Resp.
P1	0.98	0.00	0.78	1.81	0.27	0.64	0.55	0.38	0.31	0.27	0.95	0.00
P2	0.13	1.71	0.79	1.71	1.64	1.14	1.71	1.14	0.98	0.50	1.41	0.13
P3	0.57	2.41	1.07	0.57	0.13	1.07	0.98	0.79	0.21	0.21	0.29	0.21

### 3.1 Performance contrast between learning methods

Individual batch and cohort-level transfer learning methods were compared to determine their effectiveness in improving prediction error (RMSE) and outcome correlation (r) within participants, as detailed in Tables 4 and 5 and as averaged across participants in Table 6. Results from individual batch and transfer incremental models revealed participant-specific heterogeneity in certain subscales. Comparing the lowest and next lowest model errors, transfer learning generally outperformed individual batch learning across most participant-scale combinations; however, there were exceptions where individual batch models provided lower prediction error (P1: walking, stairs, composite; P2: composite; P3: cutting, turning, orthopnea).

**Table 4:** Mean model error (RMSE) across pseudo-label interpolations for each learning method.

Pt.	Domain	ALSFRS-R	Individual batch	Transfer batch	Transfer incremental
			RMSE (95% CI)	RMSE (95% CI)	RMSE (95% CI)
1		Speech	0.20 (0.14–0.28)	0.08 (0.07–0.10)${}^{a}$	**0.04** (0.03–0.04)${}^{a,b}$
	Bulbar	Salivation	—	—	—
		Swallowing	0.34 (0.19–0.49)	**0.21** (0.11–0.29)	0.23 (0.17–0.28)
		Handwriting	0.37 (0.20–0.54)	0.35 (0.12–0.47)	**0.15** (0.11–0.17)${}^{a}$
	Fine motor	Cutting	0.14 (0.10–0.18)	**0.09** (0.07–0.12)	0.12 (0.03–0.16)
		Dressing	0.29 (0.22–0.33)	**0.27** (0.21–0.31)	0.35 (0.28–0.42)
		Turning	0.28 (0.23–0.32)	0.32 (0.29–0.35)	**0.26** (0.24–0.28)${}^{b}$
	Gross motor	Walking	**0.05** (0.02–0.12)${}^{b,c}$	0.25 (0.16–0.29)	0.26 (0.15–0.32)
		Stairs	**0.00** (0.00–0.01)${}^{b,c}$	0.04 (0.04–0.04)	0.08 (0.08–0.08)
		Dyspnea	—	0.50 (0.41–0.55)	**0.42** (0.30–0.49)
	Respiratory	Orthopnea	0.48 (0.45–0.52)	0.12 (0.11–0.14)${}^{a}$	**0.11** (0.11–0.13)${}^{a}$
		Respiratory	—	—	—
	Composite	Composite	**2.76** (1.69–4.72)	3.29 (2.15–4.21)	3.24 (3.02–3.39)
2		Speech	–	0.27 (0.20–0.30)	**0.26** (0.20–0.29)
	Bulbar	Salivation	0.49 (0.45–0.52)	0.21 (0.17–0.27)${}^{a}$	**0.20** (0.16–0.28)${}^{a}$
		Swallowing	0.17 (0.15–0.18)	**0.04** (0.04–0.05)${}^{a}$	**0.04** (0.04–0.05)${}^{a}$
		Handwriting	0.45 (0.34–0.51)	0.12 (0.08–0.15)${}^{a}$	**0.10** (0.09–0.12)${}^{a}$
	Fine motor	Cutting	0.36 (0.30–0.43)	0.08 (0.06–0.11)${}^{a}$	**0.05** (0.03–0.07)${}^{a}$
		Dressing	0.35 (0.31–0.38)	0.11 (0.10–0.12)${}^{a}$	**0.06** (0.04–0.08)${}^{a,b}$
		Turning	0.30 (0.30–0.30)	**0.03** (0.02–0.05)${}^{a,c}$	0.12 (0.08–0.14)${}^{a}$
	Gross motor	Walking	0.30 (0.26–0.33)	0.14 (0.08–0.19)${}^{a}$	**0.06** (0.05–0.07)${}^{a,b}$
		Stairs	0.30 (0.21–0.36)	0.24 (0.24–0.25)	**0.23** (0.23–0.24)
		Dyspnea	—	**0.46** (0.38–0.51)	0.54 (0.44–0.63)
	Respiratory	Orthopnea	0.34 (0.29–0.42)	0.31 (0.30–0.32)	**0.30** (0.29–0.33)
		Respiratory	—	**0.30** (0.19–0.40)	0.31 (0.19–0.40)
	Composite	Composite	**3.52** (3.01–4.41)${}^{b}$	6.03 (4.86–8.28)	4.41 (3.92–4.66)${}^{b}$
3		Speech	0.38 (0.21–0.49)	**0.15** (0.13–0.19)${}^{a}$	0.19 (0.17–0.21)${}^{a}$
	Bulbar	Salivation	0.20 (0.10–0.39)	**0.15** (0.11–0.20)${}^{c}$	0.34 (0.30–0.36)
		Swallowing	0.12 (0.00–0.24)	**0.10** (0.09–0.10)${}^{c}$	0.16 (0.15–0.18)
		Handwriting	0.12 (0.00–0.18)	**0.06** (0.05–0.06)${}^{c}$	0.10 (0.08–0.10)
	Fine motor	Cutting	**0.02** (0.01–0.02)${}^{c}$	**0.02** (0.02–0.02)	0.03 (0.02–0.03)
		Dressing	0.35 (0.18–0.46)	0.16 (0.13–0.18)	**0.15** (0.12–0.16)${}^{a}$
		Turning	**0.15** (0.07–0.20)	0.16 (0.15–0.18)${}^{c}$	0.22 (0.18–0.29)
	Gross motor	Walking	0.39 (0.22–0.51)	0.17 (0.15–0.20)${}^{a}$	**0.16** (0.14–0.20)${}^{a}$
		Stairs	0.09 (0.00–0.16)	**0.03** (0.02–0.05)${}^{c}$	0.19 (0.06–0.26)
		Dyspnea	0.20 (0.20–0.20)${}^{c}$	**0.14** (0.10–0.21)${}^{c}$	0.36 (0.32–0.39)
	Respiratory	Orthopnea	**0.10** (0.00–0.18)	0.12 (0.05–0.16)	0.22 (0.11–0.32)
		Respiratory	0.22 (0.22–0.22)	0.16 (0.11–0.22)	**0.12** (0.09–0.16)${}^{a}$
	Composite	Composite	3.17 (2.27–4.62)	3.01 (2.60–3.67)	**2.97** (2.21–4.44)

Best model for each column-wise comparison per row is provided in bold.

${}^{a}$Significantly better than Individual Batch (p<0.05).

${}^{b}$Significantly better than Transfer Batch (p<0.05).

${}^{c}$Significantly better than Transfer Incremental (p<0.05).

**Table 5:** Mean model outcome correlation (r) across pseudo-label interpolations for each learning method.

Pt.	Domain	ALSFRS-R	Individual batch	Transfer batch	Transfer incremental
			r (95% CI)	r (95% CI)	r (95% CI)
1		Speech	−0.05 (−0.15 to 0.00)	−0.02 (−0.04 to 0.00)	**0.22** (0.00 to 0.66)
	Bulbar	Salivation	—	—	—
		Swallowing	−0.05 (−0.22 to 0.18)	**0.80** (0.75 to 0.84)${}^{a}$	0.62 (0.15 to 0.86)
		Handwriting	−0.07 (−0.20 to 0.00)	−0.02 (−0.06 to 0.00)	**0.08** (0.00 to 0.25)
	Fine motor	Cutting	0.00 (0.00 to 0.00)	0.00 (0.00 to 0.00)	0.00 (0.00 to 0.00)
		Dressing	**0.53** (0.33 to 0.68)${}^{c}$	0.40 (−0.13 to 0.85)	−0.20 (−0.29 to −0.06)
		Turning	−0.07 (−0.41 to 0.28)	−0.03 (−0.11 to 0.05)	**0.09** (−0.01 to 0.16)
	Gross motor	Walking	0.10 (0.00 to 0.31)	**0.24** (0.00 to 0.73)	**0.24** (0.00 to 0.73)
		Stairs	0.00 (0.00 to 0.00)	0.00 (0.00 to 0.00)	0.00 (0.00 to 0.00)
		Dyspnea	—	0.23 (−0.20 to 0.51)	**0.75** (0.60 to 0.85)${}^{b}$
	Respiratory	Orthopnea	0.30 (0.20 to 0.38)	0.98 (0.97 to 0.99)${}^{a}$	**0.99** (0.99 to 0.99)${}^{a}$
		Respiratory	—	—	—
	Composite	Composite	0.03 (−0.07 to 0.10)	−0.04 (−0.49 to 0.74)	**0.57** (0.50 to 0.71)${}^{a}$
2		Speech	—	0.56 (0.46 to 0.72)	**0.75** (0.72 to 0.78)${}^{b}$
	Bulbar	Salivation	0.09 (−0.13 to 0.36)	**−0.28** (−0.40 to −0.13)	−0.20 (−0.35 to −0.10)
		Swallowing	0.00 (0.00 to 0.00)	0.00 (0.00 to 0.00)	0.00 (0.00 to 0.00)
		Handwriting	0.27 (−0.01 to 0.56)	0.32 (0.00 to 0.62)	**0.46** (0.14 to 0.84)
	Fine motor	Cutting	0.15 (−0.13 to 0.52)	−0.22 (−0.48 to 0.01)	**0.28** (−0.07 to 0.60)
		Dressing	0.21 (0.03 to 0.46)	0.94 (0.92 to 0.96)${}^{a}$	**0.99** (0.98 to 0.99)a,b
		Turning	0.21 (0.07 to 0.45)	0.24 (−0.14 to 0.45)	**0.67** (0.52 to 0.91)a,b
	Gross motor	Walking	0.20 (0.05 to 0.31)	0.93 (0.87 to 0.96)${}^{a}$	**0.98** (0.96 to 0.99)${}^{a}$
		Stairs	0.46 (0.32 to 0.65)	0.88 (0.80 to 0.93)${}^{a}$	**0.92** (0.89 to 0.94)${}^{a}$
		Dyspnea	—	0.88 (0.84 to 0.90)	**0.94** (0.92 to 0.95)${}^{b}$
	Respiratory	Orthopnea	0.55 (0.50 to 0.62)	0.67 (0.56 to 0.84)	**0.73** (0.66 to 0.87)${}^{a}$
		Respiratory	—	0.28 (−0.26 to 0.61)	**0.48** (0.31 to 0.64)
	Composite	Composite	0.48 (0.34 to 0.66)${}^{b}$	0.11 (−0.12 to 0.23)	**0.63** (0.61 to 0.68)${}^{b}$
3		Speech	**−0.13** (−0.23 to −0.04)	0.00 (−0.57 to 0.31)	0.04 (−0.41 to 0.26)
	Bulbar	Salivation	**−0.06** (−0.22 to 0.22)	0.00 (−0.01 to 0.00)	0.03 (0.00 to 0.08)
		Swallowing	0.00 (0.00 to 0.00)	0.00 (0.00 to 0.00)	0.00 (0.00 to 0.00)
		Handwriting	0.00 (0.00 to 0.00)	0.00 (0.00 to 0.00)	0.00 (0.00 to 0.00)
	Fine motor	Cutting	0.00 (0.00 to 0.00)	0.00 (0.00 to 0.00)	0.00 (0.00 to 0.00)
		Dressing	−0.20 (−0.35 to 0.03)	0.31 (0.02 to 0.75)	**0.37** (0.09 to 0.79)${}^{a}$
		Turning	−0.08 (−0.25 to 0.00)	0.08 (0.00 to 0.24)	**0.10** (0.00 to 0.32)
	Gross motor	Walking	−0.18 (−0.25 to −0.08)	**−0.38** (−0.43 to −0.33)${}^{a}$	−0.37 (−0.43 to −0.32)${}^{a}$
		Stairs	0.00 (0.00 to 0.00)	0.00 (0.00 to 0.00)	0.00 (0.00 to 0.00)
		Dyspnea	−0.30 (−0.30 to −0.30)${}^{b}$	−0.03 (−0.09 to 0.07)	**0.47** (0.18 to 0.63)${}^{a,b}$
	Respiratory	Orthopnea	0.39 (0.00 to 0.59)	0.60 (0.00 to 0.94)	**0.64** (0.00 to 0.96)
		Respiratory	−0.31 (−0.31 to −0.31)	−0.27 (−0.32 to −0.20)	**0.79** (0.76 to 0.83)${}^{a,b}$
	Composite	Composite	−0.18 (−0.29 to −0.04)	**−0.54** (−0.58 to −0.50)${}^{a}$	−0.41 (−0.69 to 0.08)

Best model for each column-wise comparison per row is provided in bold.

${}^{a}$Significantly better than individual batch (p<0.05).

${}^{b}$Significantly better than transfer batch (p<0.05).

${}^{c}$Significantly better than transfer incremental (p<0.05).

**Table 6:** Mean model prediction error (RMSE) and outcome correlation (r) across pseudo-label interpolations and participants for each learning method.

Domain	ALSFRS-R	Individual batch	Transfer batch	Transfer incremental
		RMSE (95% CI)	RMSE (95% CI)	RMSE (95% CI)
	Speech	0.29 (0.14 to 0.44)	**0.16** (0.10 to 0.24)	**0.16** (0.08 to 0.24)
Bulbar	Salivation	0.35 (0.14 to 0.55)	**0.18** (0.12 to 0.24)	0.27 (0.18 to 0.36)
	Swallowing	0.21 (0.10 to 0.31)	**0.12** (0.05 to 0.19)	0.14 (0.08 to 0.21)
	Handwriting	0.31 (0.17 to 0.46)	0.17 (0.04 to 0.30)	**0.12** (0.09 to 0.14)${}^{a}$
Fine motor	Cutting	0.19 (0.06 to 0.32)	**0.07** (0.04 to 0.10)	**0.07** (0.02 to 0.12)
	Dressing	0.33 (0.26 to 0.40)	**0.18** (0.12 to 0.24)${}^{a}$	**0.18** (0.08 to 0.29)
	Turning	0.24 (0.18 to 0.30)	**0.17** (0.07 to 0.27)	0.20 (0.14 to 0.26)
Gross motor	Walking	0.25 (0.12 to 0.38)	0.19 (0.14 to 0.24)	**0.16** (0.08 to 0.24)
	Stairs	0.13 (0.03 to 0.27)	**0.11** (0.02 to 0.20)	0.17 (0.11 to 0.26)
	Dyspnea	**0.20** —	0.36 (0.22–0.50)	0.44 (0.36–0.52)
Respiratory	Orthopnea	0.31 (0.17–0.45)	**0.18** (0.11–0.26)	0.22 (0.14–0.29)
	Respiratory	**0.22** –	0.23 (0.11–0.35)	**0.22** (0.08–0.35)
Subscale	Mean	0.25 (0.22–0.29)	**0.18** (0.14–0.22)	0.20 (0.14–0.25)
	Composite	**3.15** (2.24–4.05)	4.11 (2.69–5.53)	3.54 (2.81–4.27)
		r (95% CI)	r (95% CI)	r (95% CI)
	Speech	−0.09 (−0.19 to 0.01)	0.18 (−0.11 to 0.48)	**0.34** (0.01–0.66)${}^{a}$
Bulbar	Salivation	0.02 (−0.22 to 0.26)	**−0.14** (−0.32 to 0.04)	−0.08 (−0.24 to 0.08)
	Swallowing	−0.02 (−0.10 to 0.07)	**0.27** (−0.04 to 0.58)	0.21 (−0.08 to 0.50)
	Handwriting	0.07 (−0.10 to 0.23)	0.10 (−0.07 to 0.28)	**0.18** (−0.04 to 0.40)
Fine motor	Cutting	0.06 (−0.11 to 0.22)	−0.07 (−0.23 to 0.06)	**0.09** (−0.09 to 0.29)
	Dressing	0.18 (−0.10 to 0.46)	**0.55** (0.22–0.88)	0.39 (−0.04 to 0.81)
	Turning	0.02 (−0.18 to 0.22)	0.10 (−0.07 to 0.26)	**0.29** (0.04–0.53)
Gross motor	Walking	0.04 (−0.12 to 0.20)	0.26 (−0.20 to 0.73)	**0.28** (−0.20 to 0.76)
	Stairs	0.17 (−0.04 to 0.39)	0.33 (−0.05 to 0.71)	**0.35** (−0.05 to 0.74)
	Dyspnea	−0.30 (—)	0.36 (0.01 to 0.70)	**0.72** (0.52 to 0.91)
Respiratory	Orthopnea	0.41 (0.25 to 0.57)	0.75 (0.50 to 1.00)	**0.79** (0.54 to 1.03)
	Respiratory	−0.31 (—)	0.01 (−0.44 to 0.45)	**0.64** (0.42 to 0.84)
Subscale	Mean	0.02 (−0.09 to 0.13)	0.22 (0.08 to 0.37)	**0.35** (0.20 to 0.49)
	Composite	0.11 (−0.13 to 0.35)	−0.16 (−0.51 to 0.20)	**0.27** (−0.16 to 0.69)

Best model for each column-wise comparison per row is provided in bold.

${}^{a}$Significantly better than individual batch (p<0.05).

#### 3.1.1 Mean performance across interpolations

The bulbar ALSFRS-R scales (speech, salivation, swallowing) demonstrated cohort-level trends, with both transfer learning models outperforming individual batch models. For speech, transfer incremental learning led to improved mean prediction error and correlation compared to transfer batch learning in P1 (RMSE =0.08→0.04∗, r=−0.02→0.22) and P2 (RMSE =0.27→0.26, r=0.56→0.75∗), while P3 showed an increase in mean prediction error and a slight change in correlation (RMSE =0.15→0.19, r=0→0.04), indicating that speech function has shared cohort-level substrates, with participant-specific changes better captured by transfer incremental fine-tuning than by individual or transfer batch models. For salivation, transfer incremental learning resulted in a negligible change in mean prediction error in P2 (RMSE =0.21→0.20), while the error increased in P3 (RMSE =0.15∗→0.34) compared to transfer batch learning. P2 showed a minor decrease in prediction correlation (r=−0.28→−0.20) with a slight change in P3 (r=0→0.03), suggesting slight participant heterogeneity in salivation decline. For swallowing, transfer incremental fine-tuning increased the prediction error with decreased correlation for P1 (RMSE =0.21→0.23, r=0.80→0.62) and with no change in correlation for P3 [RMSE =0.10∗→0.16, r=0 (0–0)]. P2 showed no change in error or correlation [RMSE =0.04 (0.04–0.05), r=0 (0–0)].

Fine-motor subscales (handwriting, cutting, and dressing) showed variable responses to individual batch and transfer incremental fine-tuning across participants, indicating an overall weak participant-level heterogeneity. The fact that transfer learning models outperformed individual batch models implies that fine-motor decline progression patterns are relatively homogeneous across the cohort. For handwriting, transfer incremental fine-tuning improved mean prediction error and correlation for P1 (RMSE =0.35→0.15, r=−0.02→0.08) and P2 (RMSE =0.12→0.10, r=0.32→0.46), whereas P3 exhibited a slight increase in prediction error (RMSE =0.06∗→0.10) with no change in correlation [r=0(0–0)]. In contrast, the cutting subscale exhibited more cohort homogeneity, as only P2 showed improvement with transfer incremental learning (RMSE =0.08→0.05, r=−0.22→0.28), while P1 (RMSE =0.09→0.12) and P3 (RMSE =0.02→0.03) demonstrated increased prediction error without correlation improvement [r=0(0–0)] compared to the transfer batch model. The dressing subscale showed a moderate level of heterogeneity between participants and the cohort, with slight improvements from transfer incremental learning in mean RMSE and r for P2 (RMSE =0.11→0.06∗, r=0.94→0.99∗) and P3 (RMSE =0.16→0.15, r=0.31→0.37). Conversely, both error and correlation worsened for P1 (RMSE =0.27→0.35, r=0.40→−0.20).

For the gross motor ALSFRS-R scales (turning, walking, stairs), individual batch models outperformed transfer batch models in a few instances, indicating heterogeneity in these cases. Individual batch learning demonstrated lower error but with a minor correlation decrease in the turning subscale for P3 (RMSE =0.15→0.16, r=−0.08→0.08) compared to transfer batch. Similarly, individual batch models had better error with lower correlation than transfer batch learning in the walking subscale for P1 (RMSE =0.05∗→0.25, r=0.10→0.24) and a marginal improvement in stairs for P1 [RMSE =0∗→0.04, r=0(0–0)]. For turning, transfer incremental improved both error and correlation in P1 (RMSE =0.32→0.26∗, r=−0.03→0.09) and increased both error and correlation in P2 (RMSE =0.03∗→0.12, r=0.24→0.67∗) compared to transfer batch. The walking subscale also showed mixed results between the transfer methods. Transfer incremental learning improved both mean error and correlation over transfer batch for P2 (RMSE =0.14→0.06∗, r=0.93→0.98) and only slightly improved for P3 (RMSE =0.17→0.16, r=−0.38→−0.37) but with a minor error increase with no change in correlation for P1 (RMSE =0.25→0.26, r=0.24). For stairs, transfer batch and incremental models showed a marginal difference in error and increased correlation for P2 (RMSE =0.24→0.23, r=0.88→0.92), while the model error increased with transfer incremental learning for P3 [RMSE =0.03∗→0.19, r=0(0–0)].

Within the respiration-related ALSFRS-R scales (dyspnea, orthopnea, respiratory), the collected orthopnea and respiratory scores for P1 and P2 exhibited low variance, as presented in Table 3, which prevented the fitting of individual batch models for those participants. For dyspnea, transfer incremental learning significantly improved correlation for all participants and reduced prediction error for P1 (RMSE =0.50→0.42, r=0.23→0.75); however, it increased the error for P2 (RMSE =0.46→0.54, r=0.88→0.94) and P3 (RMSE =0.14∗→0.36, r=−0.03→0.47). For orthopnea, transfer incremental models resulted in marginal differences in prediction error and a slight improvement in correlation for P1 (RMSE =0.12→0.11, r=0.98→0.99) and P2 (RMSE =0.31→0.30, r=0.67→0.73). However, individual batch models had slightly better error but lower correlation than transfer batch models in P3 (RMSE =0.10→0.12, r=0.39→0.60), indicating that the P3 orthopnea trajectory was not fully captured by the shared model, pointing toward participant heterogeneity, given the small improvements seen in P1 and P2 and the benefit P3 showed from individual batch learning. Similar to orthopnea, respiratory function also displayed participant heterogeneity, with transfer incremental learning resulting in a slight change in error but an increase in correlation between transfer batch and incremental learning for P2 (RMSE =0.30→0.31, r=0.28→0.48), while P3 had improved error and a significant increase in correlation (RMSE =0.16→0.12, r=−0.27→0.79).

To summarize overall functional status across bulbar, fine motor, gross motor, and respiratory items, the composite ALSFRS-R scale captures decline trends as a single index. For the composite ALSFRS-R scale, individual batch learning models resulted in a lower mean prediction error compared to transfer batch learning, with a marginal difference in outcome correlation for P1 (RMSE =2.76→3.29, r=0.03→−0.04) and with significant improvements in error and correlation for P2 (RMSE =3.52∗→6.03, r=0.48∗→0.11), demonstrating that modeled composite scores are mostly participant-specific. Transfer incremental learning slightly improved prediction error and decreased correlation for P3 (RMSE =3.01→2.97, r=−0.54→−0.41) over transfer batch models. This performance was also comparable to individual batch learning [RMSE =3.17 (2.27–4.62), r=−0.18 (−0.29–−0.04)].

#### 3.1.2 Mean group-level performance across interpolations

Contrasting the mean model performances across participants and learning methods provided evidence for whether functional domains exhibit patient-specific heterogeneity or cohort-level homogeneity. Bulbar area functions demonstrated mixed patterns, with speech exhibiting a combination of cohort homogeneity and participant heterogeneity. This was evidenced by transfer incremental learning improving outcome error and correlation over individual batch models (RMSE ≈0.29→0.16, r≈−0.09→0.34∗). In contrast, salivation showed near-zero correlation for the individual model and persistent negative correlations for transfer models, suggesting high noise or complex participant-level patterns. Swallowing showed more cohort homogeneity, with transfer batch models [RMSE ≈0.12 (0.05–0.19), r≈0.27 (−0.04–0.58)] outperforming both individual batch and incremental fine-tuned models. Fine motor area functions similarly demonstrated mixed results, as handwriting exhibited cohort patterns benefiting from transfer learning and participant heterogeneity, with incremental fine-tuning improving error and correlation over individual batch models (RMSE ≈0.31→0.12∗, r≈0.07→0.18). Cutting displayed similar heterogeneity, with incremental fine-tuning improving correlation performance over transfer batch models (r≈−0.07→0.09) with nearly equal prediction error. Dressing had moderate cohort homogeneity, with transfer batch showing a stronger correlation than incremental fine-tuning (r≈0.55→0.39) with no change in error. The gross motor area functions were predominantly cohort homogeneous but showed selective participant heterogeneity, particularly in walking, where incremental fine-tuning marginally improved error and correlation over fine-tuning (RMSE ≈0.19→0.16, r≈0.26→0.28), and in stairs, where incremental fine-tuning improved both error and correlation over individual batch models (RMSE ≈0.13→0.17, r≈0.17→0.33). These results indicate that participant variations exist within the predominant cohort patterns. Respiratory functions exhibited the strongest evidence of patient heterogeneity, with incremental fine-tuning of transfer models improving negative correlations from individual batch in dyspnea (r≈−0.30→0.72), orthopnea (r≈0.41→0.79), and respiratory (r≈−0.31→0.64).

#### 3.1.3 Mean group-level performance across interpolations and ALSFRS-R scales

As shown in the Subscale Mean row of Table 6, transfer batch models demonstrated the best subscale performance with the lowest mean prediction error [RMSE ≈0.18 (0.14–0.22)], outperforming transfer incremental [RMSE ≈0.20 (0.14–0.25)] and individual batch learning (RMSE ≈0.25 (0.22–0.29)]. Overall, across all participants and interpolation techniques, transfer incremental learning showed the highest mean correlation [r≈0.35 (0.20–0.49)] compared to transfer batch [r≈0.22 (0.08–0.37)] and individual batch learning (r≈0.02 (−0.09–0.13)] for subscale prediction. On composite scales, individual batch models showed the lowest error [RMSE ≈3.15 (2.24–4.05)] but only weak correlation [r≈0.11 (−0.13–0.35)], while transfer batch resulted in the highest error [RMSE ≈4.11 (2.69–5.53)] and a negative correlation [r≈−0.16 (−0.51–0.20)]. Transfer incremental models had the best balance between error and correlation for the composite scale, with a moderate error (RMSE ≈3.54 (2.81–4.27)], slightly higher than the error from individual batch models but lower than transfer batch models, and improved correlation [r≈0.27 (−0.16–0.69)].

### 3.2 Performance contrast between pseudo-label interpolations

To evaluate how the pseudo-labeling interpolation approach affects model performance, prediction error and outcome correlation were averaged across learning methods, as detailed in Tables 7 and 8 and across participants in Table 9. Results demonstrate that the non-linear cubic polynomial and self-attention interpolation of ALSFRS-R scales follow more closely with daily changes in in-home sensor health measurements, with a few exceptions where linear interpolation resulted in lower errors (P1: composite; P2: dressing, orthopnea, composite; P3: turning, respiratory, composite), as illustrated by the Taylor diagrams in Figure 5.

**Table 7:** Mean model prediction error (RMSE) across learning methods for each pseudo-labeling interpolation.

Pt.	Domain	ALSFRS-R	Linear slope	Cubic polynomial	Self-attention
			RMSE (95% CI)	RMSE (95% CI)	RMSE (95% CI)
1		Speech	0.14 (0.04–0.28)	0.09 (0.04–0.14)	0.10 (0.03–0.18)
	Bulbar	Salivation	—	—	—
		Swallowing	0.27 (0.24–0.33)	0.36 (0.28–0.49)	**0.16** (0.11–0.19)${}^{a,b}$
		Handwriting	0.39 (0.17–0.54)	0.33 (0.17–0.47)	**0.14** (0.11–0.20)
	Fine motor	Cutting	0.15 (0.12–0.18)	0.12 (0.07–0.16)	**0.07** (0.03–0.10)${}^{a}$
		Dressing	0.32 (0.29–0.34)	0.32 (0.21–0.42)	**0.27** (0.22–0.31)
		Turning	0.31 (0.27–0.35)	**0.26** (0.23–0.32)	0.28 (0.27–0.29)
	Gross motor	Walking	0.21 (0.02–0.32)	0.21 (0.02–0.32)	**0.14** (0.12–0.16)
		Stairs	**0.04** (0.01–0.08)	**0.04** (0.00–0.08)	—
		Dyspnea	0.51 (0.49–0.54)	0.51 (0.47–0.55)	**0.36** (0.30–0.41)${}^{a,b}$
	Respiratory	Orthopnea	0.26 (0.11–0.52)	**0.22** (0.11–0.47)	0.23 (0.12–0.45)
		Respiratory	—	—	—
	Composite	Composite	2.79 (1.86–3.50)	**2.38** (1.69–3.31)${}^{c}$	4.11 (3.39–4.72)
2		Speech	0.30 (0.29–0.30)	0.30 (0.29–0.30)	**0.20** (0.20–0.20)${}^{a,b}$
	Bulbar	Salivation	0.29 (0.16–0.52)	**0.28** (0.16–0.51)	0.33 (0.27–0.45)
		Swallowing	**0.08** (0.04–0.17)	0.09 (0.04–0.18)	0.09 (0.04–0.15)
		Handwriting	0.23 (0.08–0.51)	0.25 (0.12–0.50)	**0.20** (0.10–0.34)
	Fine Motor	Cutting	0.15 (0.03–0.37)	0.20 (0.07–0.43)	**0.14** (0.05–0.30)
		Dressing	**0.16** (0.05–0.31)	0.17 (0.04–0.38)	0.18 (0.08–0.36)
		Turning	0.15 (0.03–0.30)	0.16 (0.05–0.30)	**0.13** (0.02–0.30)
	Gross motor	Walking	0.17 (0.05–0.31)	0.19 (0.07–0.33)	**0.14** (0.07–0.26)
		Stairs	0.28 (0.24–0.36)	0.27 (0.24–0.33)	**0.22** (0.21–0.24)${}^{a,b}$
		Dyspnea	0.55 (0.48–0.63)	0.52 (0.51–0.54)	**0.41** (0.38–0.44)${}^{a,b}$
	Respiratory	Orthopnea	**0.29** (0.29–0.30)${}^{b,c}$	0.31 (0.31–0.31)${}^{c}$	0.36 (0.32–0.42)
		Respiratory	0.34 (0.34–0.34)${}^{b}$	0.40 (0.40–0.40)	**0.19** (0.19–0.19)${}^{a,b}$
	Composite	Composite	**4.21** (3.01–4.97)	4.22 (3.14–4.86)	5.54 (3.92–8.28)
3		Speech	0.25 (0.14–0.44)	0.26 (0.13–0.49)	**0.20** (0.19–0.21)
	Bulbar	Salivation	0.20 (0.10–0.36)	**0.19** (0.10–0.36)	0.30 (0.20–0.39)
		Swallowing	0.12 (0.09–0.15)	0.16 (0.10–0.24)	**0.09** (0.00–0.18)
		Handwriting	0.11 (0.05–0.16)	0.12 (0.06–0.18)	**0.05** (0.00–0.08)
	Fine motor	Cutting	**0.02** (0.02–0.02)	**0.02** (0.01–0.02)	—
		Dressing	0.25 (0.16–0.41)	0.27 (0.16–0.46)	**0.14** (0.12–0.18)
		Turning	**0.17** (0.15–0.19)	0.18 (0.15–0.20)	0.18 (0.07–0.29)
	Gross motor	Walking	0.24 (0.14–0.43)	0.27 (0.14–0.51)	**0.21** (0.20–0.22)
		Stairs	0.13 (0.02–0.26)	0.15 (0.03–0.26)	**0.04** (0.00–0.06)
		Dyspnea	**0.24** (0.10–0.38)	0.25 (0.11–0.39)	**0.24** (0.20–0.32)
	Respiratory	Orthopnea	0.20 (0.13–0.32)	0.19 (0.16–0.24)	**0.05** (0.00–0.11)${}^{a,b}$
		Respiratory	**0.10** (0.09–0.11)${}^{c}$	0.12 (0.10–0.14)${}^{c}$	0.20 (0.16–0.22)
	Composite	Composite	**2.38** (2.27–2.60)${}^{c}$	2.53 (2.21–2.76)${}^{c}$	4.24 (3.67–4.62)

Best model for each column-wise comparison per row is provided in bold.

${}^{a}$Significantly better than the linear slope (p<0.05).

${}^{b}$Significantly better than the cubic polynomial (p<0.05).

${}^{c}$Significantly better than self-attention (p<0.05).

**Table 8:** Mean model outcome correlation (r) across learning methods for each pseudo-labeling interpolation.

Pt.	Domain	ALSFRS-R	Linear slope	Cubic polynomial	Self-attention
			r (95% CI)	r (95% CI)	r (95% CI)
1		Speech	0.00 (0.00 to 0.00)	0.00 (0.00 to 0.00)	**0.16** (−0.15 to 0.66)
	Bulbar	Salivation	—	—	—
		Swallowing	0.49 (−0.22 to 0.86)	**0.53** (−0.11 to 0.86)	0.36 (0.15 to 0.75)
		Handwriting	0.00 (0.00 to 0.00)	0.00 (0.00 to 0.00)	0.00 (−0.20 to 0.25)
	Fine motor	Cutting	0.00 (0.00 to 0.00)	0.00 (0.00 to 0.00)	0.00 (0.00 to 0.00)
		Dressing	0.19 (−0.26 to 0.49)	**0.46** (−0.06 to 0.85)	0.09 (−0.29 to 0.68)
		Turning	−0.01 (−0.11 to 0.16)	−0.10 (−0.41 to 0.12)	**0.11** (−0.01 to 0.28)
	Gross motor	Walking	0.00 (0.00 to 0.00)	0.00 (0.00 to 0.00)	**0.59** (0.31 to 0.73)a,b
		Stairs	0.00 (0.00 to 0.00)	0.00 (0.00 to 0.00)	—
		Dyspnea	**0.68** (0.51 to 0.85)	0.59 (0.39 to 0.79)	0.20 (−0.20 to 0.60)
	Respiratory	Orthopnea	**0.79** (0.38 to 0.99)	0.76 (0.31 to 0.99)	0.73 (0.20 to 0.99)
		Respiratory	—	—	—
	Composite	Composite	0.08 (−0.36 to 0.51)	−0.02 (−0.49 to 0.50)	**0.50** (0.06 to 0.74)
2		Speech	0.59 (0.46 to 0.72)	0.63 (0.50 to 0.75)	**0.75** (0.72 to 0.78)${}^{a}$
	Bulbar	Salivation	−0.13 (−0.31 to 0.05)	**−0.30** (−0.40 to −0.13)${}^{c}$	0.04 (−0.13 to 0.36)
		Swallowing	0.00 (0.00 to 0.00)	0.00 (0.00 to 0.00)	0.00 (0.00 to 0.00)
		Handwriting	0.24 (−0.01 to 0.38)	0.13 (0.00 to 0.25)	**0.67** (0.56 to 0.84)a,b
	Fine motor	Cutting	−0.01 (−0.20 to 0.30)	0.00 (−0.07 to 0.05)	**0.21** (−0.48 to 0.60)
		Dressing	**0.80** (0.46 to 0.99)	0.69 (0.13 to 0.99)	0.64 (0.03 to 0.98)
		Turning	0.36 (0.11 to 0.57)	0.35 (0.07 to 0.52)	**0.41** (−0.14 to 0.91)
	Gross Motor	Walking	**0.75** (0.31 to 0.99)	0.70 (0.23 to 0.99)	0.66 (0.05 to 0.96)
		Stairs	0.73 (0.32 to 0.94)	0.76 (0.40 to 0.94)	**0.78** (0.65 to 0.89)
		Dyspnea	0.91 (0.88 to 0.95)	0.89 (0.84 to 0.95)	**0.92** (0.90 to 0.92)
	Respiratory	Orthopnea	0.62 (0.56 to 0.67)	0.59 (0.50 to 0.66)	**0.75** (0.53 to 0.87)
		Respiratory	**0.63** (0.61 to 0.64)b,c	0.48 (0.48 to 0.48)${}^{c}$	0.03 (−0.26 to 0.31)
	Composite	Composite	0.39 (0.21 to 0.61)	**0.43** (0.23 to 0.61)	0.41 (−0.12 to 0.68)
3		Speech	0.13 (−0.13 to 0.26)	0.11 (−0.23 to 0.31)	**−0.34** (−0.57 to −0.04)
	Bulbar	Salivation	−0.07 (−0.22 to 0.00)	−0.05 (−0.16 to 0.00)	**0.10** (−0.01 to 0.22)
		Swallowing	0.00 (0.00 to 0.00)	0.00 (0.00 to 0.00)	0.00 (0.00 to 0.00)
		Handwriting	0.00 (0.00 to 0.00)	0.00 (0.00 to 0.00)	0.00 (0.00 to 0.00)
	Fine motor	Cutting	0.00 (0.00 to 0.00)	0.00 (0.00 to 0.00)	—
		Dressing	−0.06 (−0.29 to 0.09)	0.01 (−0.35 to 0.23)	**0.52** (0.03 to 0.79)
		Turning	0.00 (0.00 to 0.00)	0.00 (0.00 to 0.00)	**0.10** (−0.25 to 0.32)
	Gross motor	Walking	−0.29 (−0.33 to −0.22)	−0.27 (−0.37 to −0.08)	**−0.37** (−0.43 to −0.25)
		Stairs	0.00 (0.00 to 0.00)	0.00 (0.00 to 0.00)	0.00 (0.00 to 0.00)
		Dyspnea	**0.35** (0.07 to 0.63)	0.25 (−0.08 to 0.59)	−0.07 (−0.30 to 0.18)
	Respiratory	Orthopnea	0.81 (0.58 to 0.95)${}^{c}$	**0.82** (0.59 to 0.96)${}^{c}$	0.00 (0.00 to 0.00)
		Respiratory	0.24 (−0.28 to 0.76)	**0.29** (−0.20 to 0.77)	0.07 (−0.32 to 0.83)
	Composite	Composite	**−0.52** (−0.69 to −0.29)	−0.46 (−0.63 to −0.20)	−0.15 (−0.50 to 0.08)

Best model for each column-wise comparison per row is provided in bold.

${}^{a}$Significantly better than the linear slope (p<0.05).

${}^{b}$Significantly better than the cubic polynomial (p<0.05).

${}^{c}$Significantly better than self-attention (p<0.05).

**Table 9:** Mean model prediction error (RMSE) and outcome correlation (r) across learning methods and participants for each pseudo-labeling interpolation.

Domain	ALSFRS-R	Linear slope	Cubic polynomial	Self-attention
		RMSE (95% CI)	RMSE (95% CI)	RMSE (95% CI)
	Speech	0.22 (0.11 to 0.33)	0.21 (0.08 to 0.33)	**0.16** (0.11 to 0.22)
Bulbar	Salivation	**0.24** (0.08 to 0.41)	**0.24** (0.06 to 0.41)	0.32 (0.22 to 0.41)
	Swallowing	0.16 (0.08 to 0.23)	0.20 (0.09 to 0.31)	**0.11** (0.06 to 0.16)
	Handwriting	0.24 (0.09 to 0.40)	0.23 (0.10 to 0.36)	**0.13** (0.06 to 0.20)
Fine motor	Cutting	0.11 (0.02 to 0.20)	0.11 (0.01 to 0.21)	**0.10** (0.00 to 0.21)
	Dressing	0.24 (0.15 to 0.34)	0.25 (0.14 to 0.37)	**0.20** (0.12 to 0.28)
	Turning	0.21 (0.13 to 0.29)	**0.20** (0.14 to 0.26)	**0.20** (0.11 to 0.28)
Gross motor	Walking	0.20 (0.10 to 0.31)	0.22 (0.11 to 0.34)	**0.16** (0.11 to 0.21)
	Stairs	0.15 (0.06 to 0.25)	0.15 (0.06 to 0.25)	**0.13** (0.02 to 0.24)
	Dyspnea	0.43 (0.24 to 0.63)	0.43 (0.25 to 0.60)	**0.32** (0.23 to 0.41)
Respiratory	Orthopnea	0.25 (0.14 to 0.35)	0.24 (0.15 to 0.34)	**0.22** (0.09 to 0.34)
	Respiratory	0.22 (0.00 to 0.44)	0.26 (0.00 to 0.52)	**0.20** (0.17 to 0.22)
Subscale	Mean	0.22 (0.18 to 0.27)	0.23 (0.18 to 0.27)	**0.19** (0.15 to 0.23)
	Composite	3.13 (2.30 to 3.95)	**3.04** (2.20 to 3.88)	4.63 (3.52 to 5.74)
		r (95% CI)	r (95% CI)	r (95% CI)
	Speech	0.20 (−0.04 to 0.43)	**0.21** (−0.07 to 0.47)	0.12 (−0.33 to 0.56)
Bulbar	Salivation	−0.10 (−0.25 to 0.05)	**−0.17** (−0.35 to 0.01)	0.07 (−0.13 to 0.27)
	Swallowing	0.16 (−0.14 to 0.47)	**0.18** (−0.12 to 0.47)	0.12 (−0.07 to 0.31)
	Handwriting	0.08 (−0.04 to 0.20)	0.04 (−0.03 to 0.12)	**0.22** (−0.06 to 0.50)
Fine motor	Cutting	0.00 (−0.11 to 0.10)	0.00 (−0.02 to 0.02)	**0.11** (−0.31 to 0.52)
	Dressing	0.31 (−0.05 to 0.67)	0.39 (0.02 to 0.75)	**0.42** (0.04 to 0.80)
	Turning	0.12 (−0.06 to 0.29)	0.08 (−0.13 to 0.29)	**0.21** (−0.06 to 0.47)
Gross motor	Walking	0.15 (−0.23 to 0.54)	0.14 (−0.23 to 0.51)	**0.29** (−0.15 to 0.74)
	Stairs	0.24 (−0.07 to 0.55)	0.25 (−0.06 to 0.57)	**0.39** (−0.06 to 0.84)
	Dyspnea	**0.65** (0.30 to 1.00)	0.58 (0.18 to 0.98)	0.29 (−0.19 to 0.77)
Respiratory	Orthopnea	**0.74** (0.57 to 0.91)	0.72 (0.53 to 0.90)	0.49 (0.15 to 0.83)
	Respiratory	**0.43** (−0.34 to 1.20)	0.38 (−0.27 to 1.03)	0.05 (−0.58 to 0.68)
Subscale	Mean	**0.25** (0.11 to 0.39)	0.23 (0.09 to 0.37)	0.24 (0.15 to 0.31)
	Composite	−0.02 (−0.38 to 0.35)	−0.02 (−0.38 to 0.35)	**0.25** (−0.10 to 0.60)

Best model for each column-wise comparison per row is provided in bold.

#### 3.2.1 Mean performance across learning methods

The bulbar ALSFRS-R scales (speech, salivation, swallowing) resulted in mean improvements to model error from non-linear cubic and self-attention interpolation compared to linear interpolation. For speech, cubic reduced model error for P1 [RMSE =0.14→0.09, r=0 (0–0)], while self-attention provided improved error and correlation for P2 (RMSE =0.30→0.20∗, r=0.59→0.75∗) and improved error and correlation for P3 (RMSE =0.25→0.20, r=0.13→−0.34). Salivation performed only marginally better with cubic interpolation over linear interpolation, with an increase in correlation for P2 (RMSE =0.29→0.28, r=−0.13→−0.30) and a minor correlation change for P3 (RMSE =0.20→0.19, r=−0.07→−0.05). For the swallowing subscale, self-attention interpolation provided lower error across all participants, with a decreased correlation for P1 (RMSE =0.27→0.16∗, r=0.49→0.36) and no change in correlation for P3 [RMSE =0.12→0.09, r=0(0–0)], while cubic and self-attention performed the same, with a very minor difference in error for P2 [RMSE =0.09→0.08, r=0(0–0)] compared to linear interpolation.

For fine-motor subscales (handwriting, cutting, and dressing), self-attention interpolation provided the lowest model error, with the exception of cutting for P3 and dressing for P2. For the handwriting scale, self-attention reduced error and either increased or maintained correlation for P1 [RMSE =0.39→0.14, r=0 (0–0)], P2 (RMSE =0.23→0.20, r=0.24→0.67∗), and P3 [RMSE =0.11→0.05, r=0 (0–0)]. Cutting also performed better on self-attention pseudo-labels, reducing error for P1 [RMSE =0.15→0.07∗, r=0 (0–0)] and for P2 (RMSE =0.15→0.14, r=−0.01→0.21) with improved correlation, while cubic and linear models performed equally for P3 [RMSE =0.02(0.01–0.02), r=0 (0–0)]. Dressing models fit on self-attention interpolated labels had decreased model error but with lower correlation for P1 (RMSE =0.32→0.27, r=0.19→0.09) and significantly better correlation for P3 (RMSE =0.25→0.14, r=−0.06→0.52), while the linear slope provided the best error and correlation compared to non-linear interpolations for P2 [RMSE =0.16 (0.05–0.31), r=0.80 (0.46–0.99)].

For gross motor ALSFRS-R scales (turning, walking, stairs), self-attention interpolation again resulted in improved model error in most cases. The turning subscale had mixed outcomes: cubic interpolation improved error over linear but with decreased correlation for P1 (RMSE =0.31→0.26, r=−0.01→−0.10) and self-attention improved error and correlation for P2 (RMSE =0.15→0.13, r=0.36→0.41) and showed a minor change in error with improved correlation for P3 (RMSE =0.17→0.18, r=0→0.10). For walking, self-attention interpolation resulted in the lowest error for all participants, with significantly improved correlation for P1 (RMSE =0.21→0.14, r=0→0.59∗), a moderate decrease in correlation for P2 (RMSE =0.17→0.14, r=0.75→0.66), and an increase for P3 (RMSE =0.24→0.21, r=−0.29→−0.37). The stairs models had the lowest prediction error with self-attention interpolation, with a slight improvement in correlation for P2 (RMSE =0.28→0.22∗, r=0.73→0.78) and no change for P3 [RMSE =0.13→0.04, r=0 (0–0)]. However, linear slope and cubic interpolation performed equally for P1 [RMSE =0.04 (0.01–0.08), r=0(0–0)].

Within the respiration-related ALSFRS-R scales (dyspnea, orthopnea, respiratory), models fit on linear slope demonstrated better error than non-linear interpolations among all functional domains. For the dyspnea scale, self-attention interpolation improved error but with a decreased correlation for P1 (RMSE =0.52→0.36∗, r=0.68→0.20) and a marginal correlation change for P2 (RMSE =0.55→0.41∗, r=0.92→0.91). Self-attention interpolation had equal prediction error to the baseline linear slope but at a decreased correlation for P3 [RMSE =0.24 (0.20–0.32), r=0.35→−0.07]. For orthopnea models, transfer batch interpolation improved error but with a slight reduction in correlation for P1 (RMSE =0.26→0.22, r=0.79→0.76) compared to the linear slope. Self-attention interpolation increased model error over linear interpolation but with improved correlation for P2 (RMSE =0.29∗→0.36, r=0.62→0.75), while the opposite occurred for P3 (RMSE =0.20→0.05∗, r=0.82∗→0), reducing model error and significantly decreasing correlation in P3. Respiratory had the lowest error from self-attention but with a significant reduction in correlation for P2 (RMSE =0.34→0.19, r=0.63∗→0.03), while the linear slope provided a slight decrease in both error and correlation compared to cubic interpolation for P3 (RMSE =0.10→0.12, r=0.24→0.29).

For the composite ALSFRS-R scale, linear slope models provided the best prediction error but had a decreased outcome correlation compared to self-attention interpolation for P1 (RMSE =2.79→4.11, r=0.08→0.50), P2 (RMSE = 4.21→5.54, r=0.39→0.41), and P3 (RMSE =2.38→4.24, r=−0.52→−0.15). The results suggest that while self-attention was best at capturing the overall changes within individual ALSFRS-R subscales, the summation of the ALSFRS-R composite score compensates these changes, resulting in a linear trajectory, confirming to clinical practice of using a linear slope to estimate the rate of functional change.

#### 3.2.2 Mean group-level performance across learning methods and ALSFRS-R scales

Self-attention interpolation had the best subscale-specific performance with the lowest mean prediction error [RMSE ≈0.19 (0.15–0.23), r≈0.24 (0.15–0.31)] across participants and ALSFRS-R subscales, as shown in the Subscale Mean row of Table 9, outperforming linear and cubic interpolation in 21 of 34 subscale comparisons excluding ties, reported in bold in Table 7. Linear [RMSE ≈0.22 (0.18–0.27), r≈0.25 (0.11–0.39)] and cubic [RMSE ≈0.23 (0.18–0.27), r≈0.23 (0.09–0.37)] interpolations showed nearly identical mean performance and were optimal in only six and five subscale models, respectively. For composite scales, the pattern reversed, with linear interpolation having lower error in two of three comparisons [RMSE ≈3.13 (2.30–3.95)] and cubic in one of three [RMSE ≈3.04 (2.20–3.88)], while self-attention raised error [RMSE ≈4.63 (3.52–5.74)], implying that composite trajectories are more accurately captured by a stable linear slope than by self-attention interpolation, which may potentially have been over-responsive. However, choice of evaluation metric also factors into pseudo-labeling selection, with self-attention providing the best correlation for composite models when prioritizing the prediction–outcome trend agreement [r≈0.25 (−0.10 to 0.60)] compared to linear and cubic interpolation [r≈−0.02 (−0.38 to 0.35)].

## 4 Discussion

Semi-supervised learning approaches were evaluated for predicting ALSFRS-R scale trajectories using in-home sensor health features. We compared participant-level batch learning and cohort-initialized transfer learning, which used batch and incremental fine-tuning strategies. The results demonstrate that adapting cohort transfer learning models with additional individual-level data through incremental fine-tuning improves prediction error (RMSE) and outcome correlation (Pearson’s r).

### 4.1 ALSFRS-R scales exhibit mixed participant-cohort homogeneity

ALS decline progression varies across different ALSFRS-R functional areas, creating multi-dimensional trajectories, where some subscales decline predictably across the cohort, while others follow patient-specific trends. As illustrated in Figure 3, rates of decline in bulbar, gross, and respiratory area measures for P1 were marked by periods of stability followed by sudden decreases compared to the regular decline observed for P2 and P3. Similarly, fine motor measures for P1 increased around November 2023 followed by a regular rate of decline, mirroring the decreases observed for P2 and P3. Cohort-level model performances, when averaged across pseudo-label interpolations, confirmed that bulbar and gross motor scales largely follow cohort-level patterns. Transfer batch learning provided the lowest error for swallowing and gross motor measures, while subscales such as speech and handwriting benefited more from transfer incremental tuning. Conversely, respiratory functions demonstrated more participant-level heterogeneity, with transfer incremental fine-tuning inverting outcome correlations from negative in individual batch models to strongly positive. These findings suggest that tailoring learning methods to the underlying homogeneity–heterogeneity profile of each functional domain will improve model optimization, with cohort-homogeneous scales making better use of transfer learning, participant-heterogeneous scales requiring incremental fine-tuning to better capture patient-specific patterns. Additionally, mixed-profile subscales could potentially benefit from adaptive learning approaches when participant-level patterns deviate from the cohort-baseline variability.

### 4.2 Transfer learning improves performance in subscale models

Incremental fine-tuning of transfer learning models provided the best balance between predictive accuracy and correlation when contrasting the results from the learning technique. The Taylor diagram analysis in Figure 6 of model predictions aggregated across participants illustrated how transfer learning models outperformed participant-level batch learning across most subscales, aligning predictions closer to reference vectors with improved accuracy and correlation. Performance differences between individual batch and transfer learning models were particularly pronounced across subscales, as presented in Table 4, where transfer batch models reduced the mean prediction error in 12 of 34 comparisons and transfer incremental learning did so in 16 of 34, excluding ties. Individual batch models exhibited increased error and weaker correlations, achieving best performance in only four ALSFRS-R scale models (P1: walking, stairs; P3: turning, orthopnea). Evaluating outcome correlation, transfer incremental learning increased correlation in 20 of 34 comparisons, as presented in Table 5. As such, although transfer incremental learning had a slightly higher mean prediction error, it was more effective at capturing individual trajectory patterns than transfer batch learning, suggesting that it is better at detecting temporal changes in ALS decline. Additionally, the improved performance of transfer learning approaches across subscales suggests that ALS progression follows cohort-level patterns predictive of individual trajectories despite disease heterogeneity, supporting the presence of underlying shared physiological or functional characteristics that are captured by sensor data as detected by cohort-level models. The effectiveness of incremental fine-tuning indicates that personalized ALS progression tracking should incorporate both group parameters and adaptive learning for predicting decline in individual patients.

### 4.3 Integrating passive sensor analytics into personalized ALS clinical care

Although preliminary and limited in generalizability, the findings from this case series suggest that integrating passive in-home sensor monitoring into routine ALS care may help clinicians better detect and anticipate functional changes between quarterly assessments, differentiating stable periods from more rapid decline. More specifically, this study shows that combining semi-supervised transfer learning with continual fine-tuning on patient-level sensor data improves the estimation of ALSFRS-R subscale trajectories compared to batch learning, supporting the use of personalized, adaptive algorithms for tracking the course of disease unique to the individual patient from models pretrained on group-level data. The improved performance of group-initialized transfer models indicates that, even with a minimal cohort of three patients, combining data across patients as a baseline model of disease progression that can be further adapted to new patients over time can leverage patterns homogeneous to the case series cohort. The findings also underscore the need for tailoring learning strategies to specific clinical problems, whether they are subscales or composite measures, to provide more reliable indicators of disease progression. In practice, clinicians could receive near real-time notifications about deviations in a patient’s functional trajectories, as this is being evaluated in the parent study. Such notifications may enable proactive adjustments to respiratory support, assistive ambulatory devices, nutritional interventions, or rehabilitation schedules, which would allow for timely interventions, rather than waiting weeks for the next in-person assessment. Sensor-based analytics may also reduce the recall bias and subjective self-assessment errors common in clinic-based evaluations while providing actionable insights into patients’ day-to-day variability, especially for those with unpredictable subscale trajectories or across ALSFRS-R functional areas. The combination of transfer and incremental learning has the potential to optimize clinical workflows and attention by identifying the specific patients, and at the proper time, who need closer monitoring and therapies. Additionally, examining modeled outcomes by the pseudo-labeling technique showed that the choice of optimal interpolation is largely outcome- and metric-specific, with performance varying across subscale and composite measures. In the context of clinical ALS trial studies, the continuous outcome estimates derived from self-supervised models could serve as prognostic endpoints.

## 5 Conclusion

This study demonstrates that semi-supervised machine learning using in-home sensor data can effectively predict ALSFRS-R scale trajectories, with incremental fine-tuned transfer learning performing well across all functional domains. Given the case series cohort (n=3), the results demonstrate feasibility and within-participant accuracy rather than generalizable effectiveness, with further confirmation requiring a larger, multi-site cohort. The findings indicate that the choice of interpolation techniques for estimating between-visit decline should be tailored to specific clinical objectives, with self-attention interpolation performing best for subscale-level monitoring and polynomial function interpolation performing better for the summated composite ALSFRS-R score. However, the generalizability of reported modeled outcomes is limited by the small participant cohort and reliance on bed sensor and motion detection data, which lack comprehensive gait measurements that may be particularly important for assessing motor function. The low prediction error-low outcome correlation models for bulbar and motor-related subscale models (P1: handwriting, cutting, stairs; P2: swallowing; P3: swallowing, handwriting, cutting, stairs) exemplify the need for motor-related measurements. Future research may be conducted to validate the learning methods applied in this analysis with a larger, multi-center study to establish broader applicability, explore complementary clinical measures such as forced volume capacity (FVC), and investigate enhanced feature engineering approaches that could improve performance for patient-heterogeneous ALSFRS-R component scales. Additionally, developing adaptive incremental learning algorithms with patient-specific clinical feedback mechanisms for ground truth scoring and extending this framework through multi-model ensemble approaches are promising directions for advancing personalized disease progression monitoring that could transform clinical decision-making in neurodegenerative care. As part of the parent study, we will expand the participant sample and train larger cohort-level transfer models with additional training data to improve their sensitivity and specificity to between-visit changes. Overall, this work aims to enable earlier detection of clinically meaningful changes in ALS progression as support for timely interventions that address functional decline.

## Data Availability

The datasets presented in this article are not readily available because they are derived from human subjects and contain sensitive information. Requests to access the datasets should be directed to William Janes, Principal Investigator, janesw@health.missouri.edu, subject to institutional review board approval.
