# Supplementary material for: Enhancing ALS progression tracking with semi-supervised ALSFRS-R scores estimated from ambient home health monitoring
Source: Front Digit Health. 2025 Sep 26;7:1657749. doi: 10.3389/fdgth.2025.1657749 (PMC12511069; doi:10.3389/fdgth.2025.1657749)
Supplement: Supplementary file 1 [file Datasheet1.pdf]

Supplementary Material

1 SUPPLEMENTARY FIGURES AND TABLES

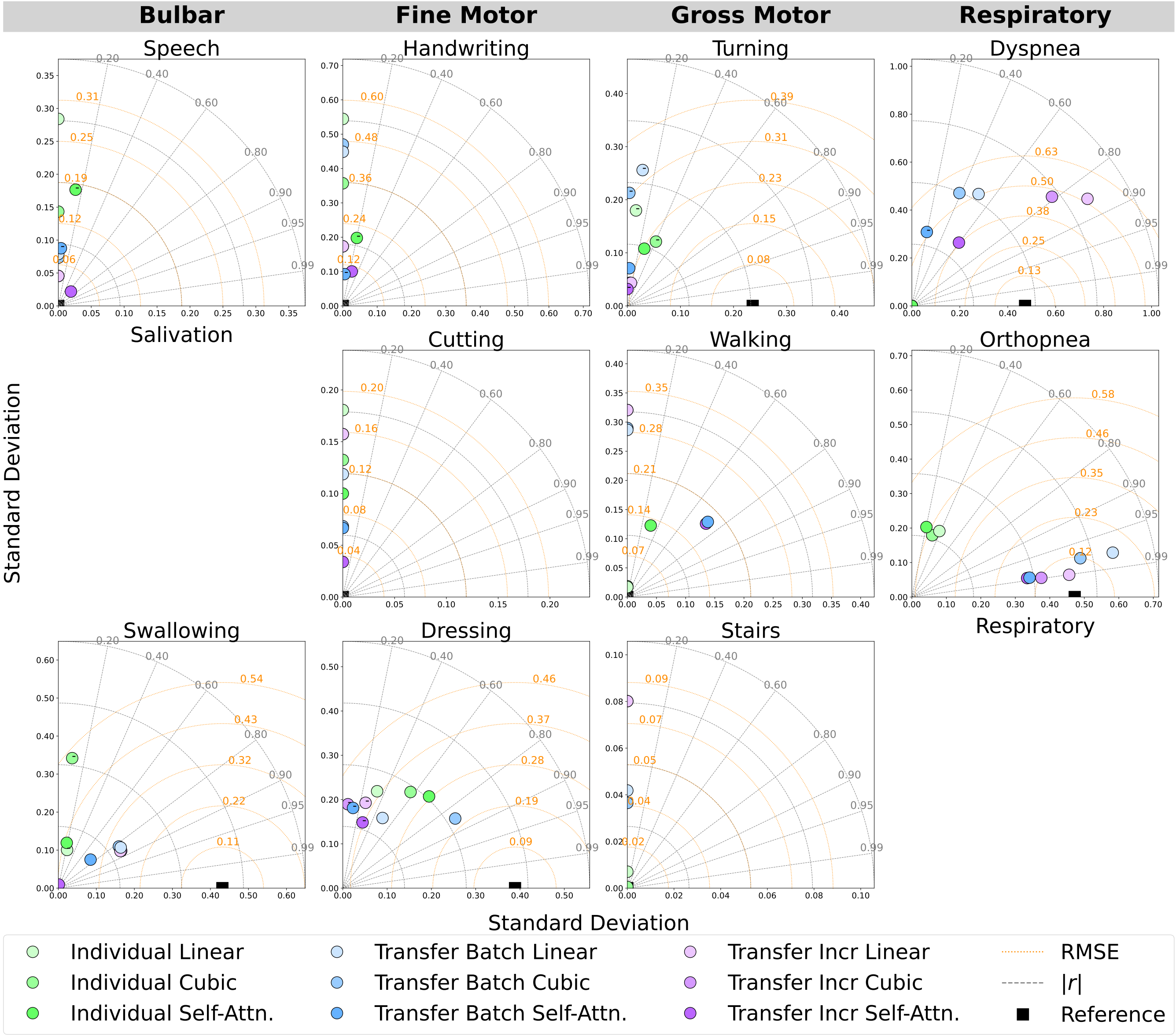

Figure S1. Participant 1 Taylor diagrams showing mean prediction error (RMSE), absolute correlation ($|r|$), and standard deviation of outcomes for each ALSFRS-R scale, annotated by negative ($-$) correlation. Salivation and Respiratory subscales were not modeled as the collected scores had variance of 0 over the study period.

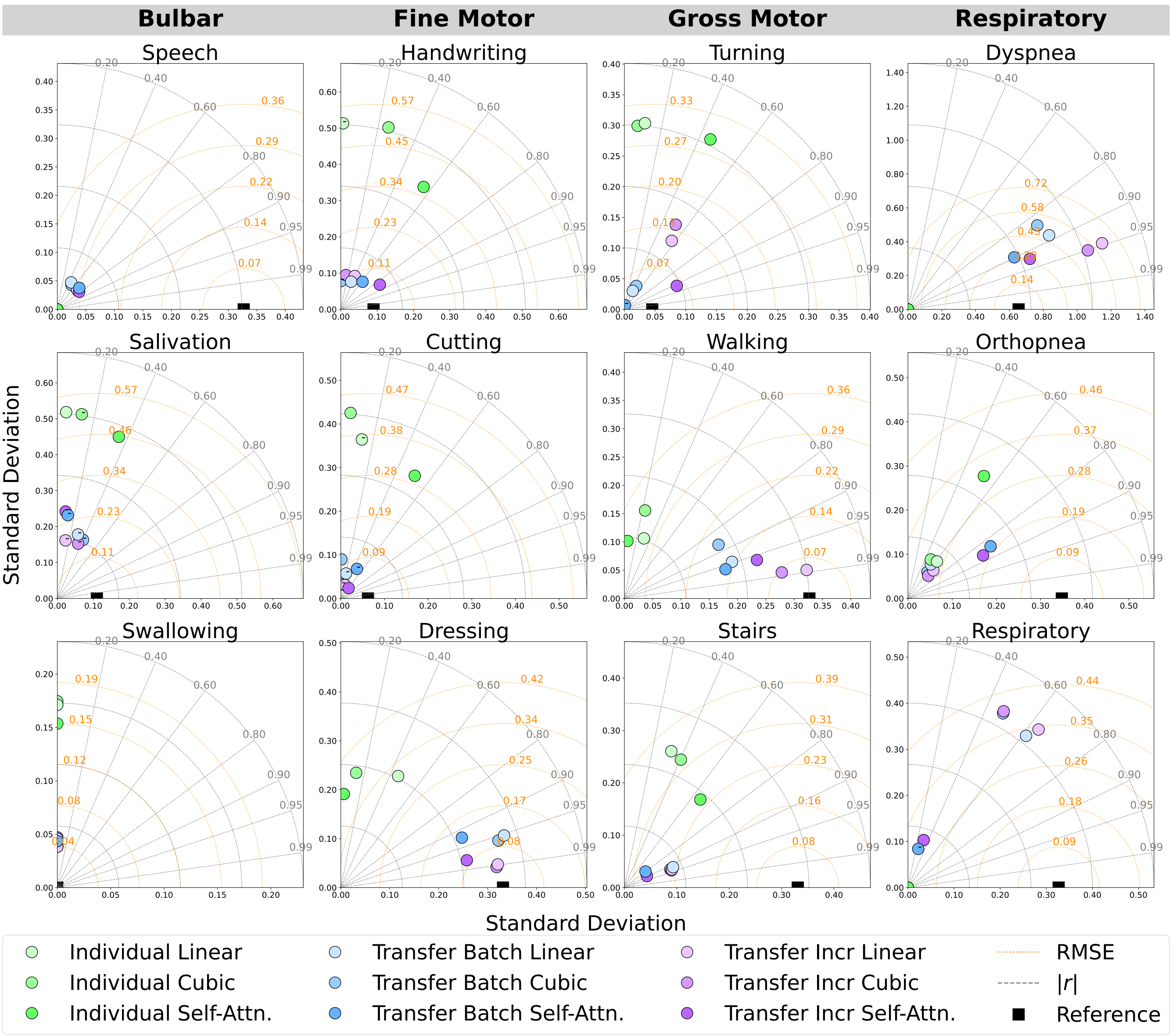

Figure S2. Participant 2 Taylor diagrams showing mean prediction error (RMSE), absolute correlation ($|r|$), and standard deviation of outcomes for each ALSFRS-R scale, annotated by negative ($-$) correlation.

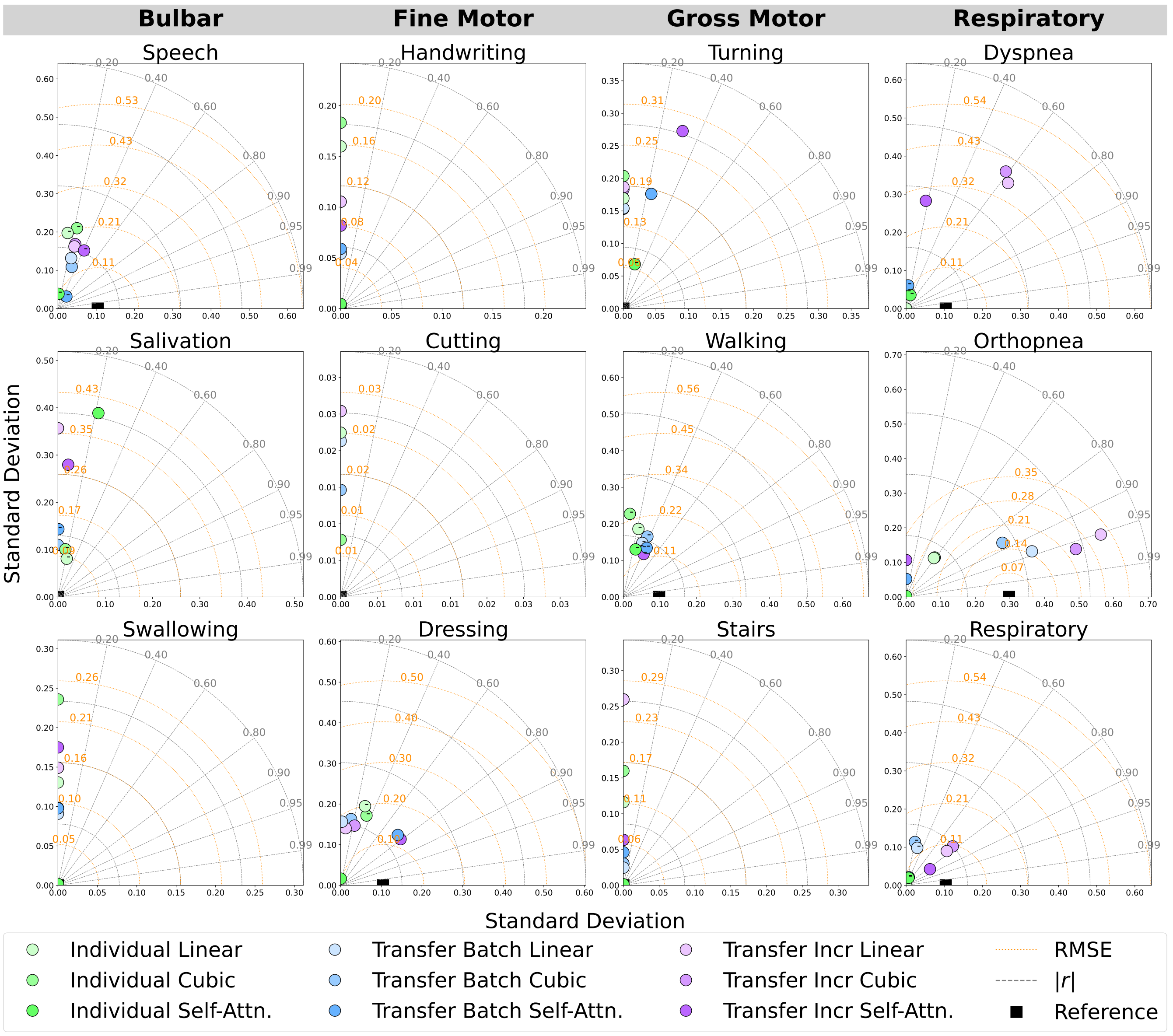

Figure S3. Participant 3 Taylor diagrams showing mean prediction error (RMSE), absolute correlation ($|r|$), and standard deviation of outcomes for each ALSFRS-R scale, annotated by negative ($-$) correlation.

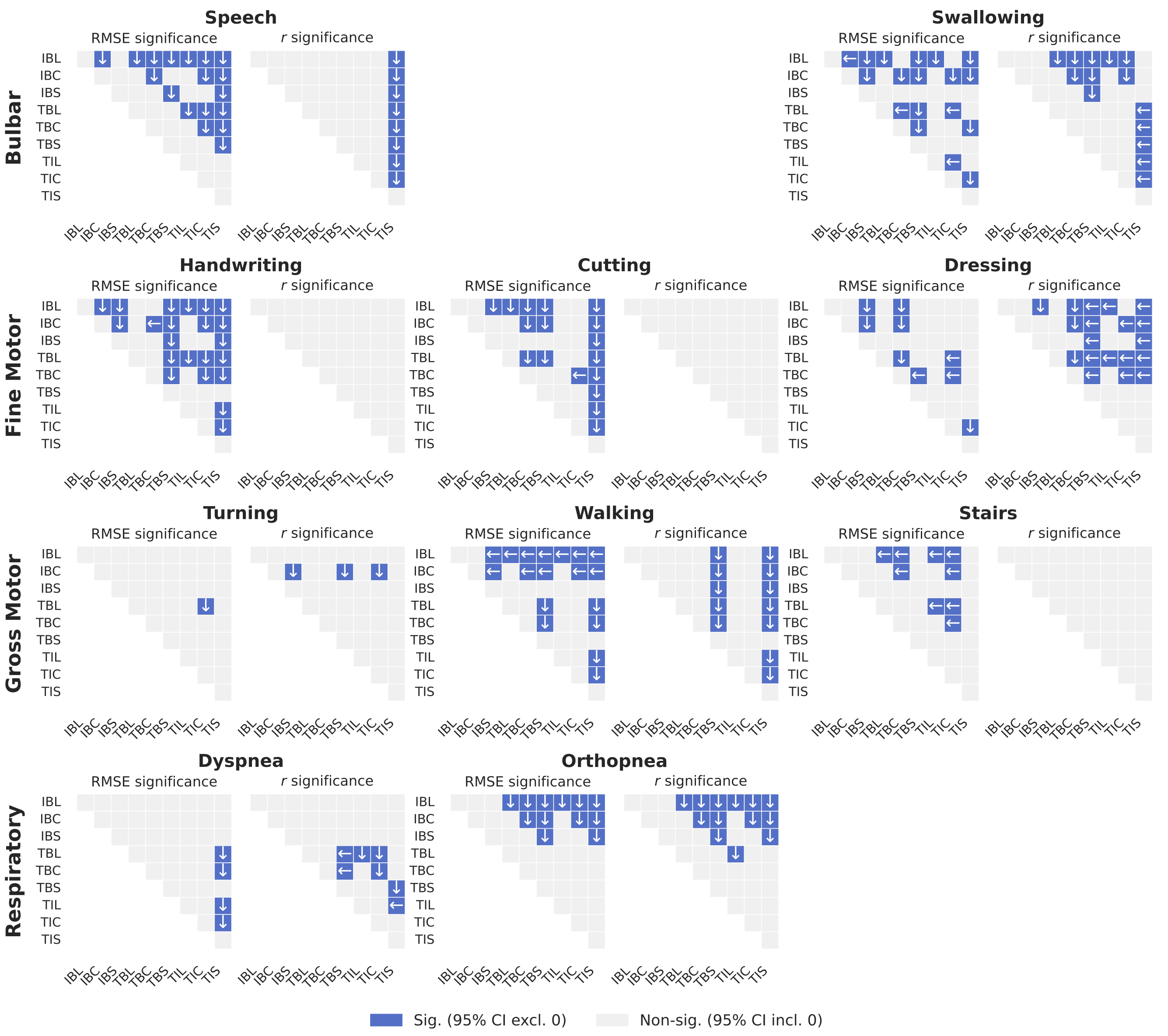

Figure S4. Participant 1 pairwise significance analysis of model performance across ALSFRS-R functional domains and targets. Each cell compares two models (IBL, IBC, IBS, TBL, TBC, TBS, TIL, TIC, TIS) for a specific item, indicating whether the difference in RMSE or r between them is statistically significant (Neutral cells: 95% confidence intervals do not overlap, Color cells: intervals overlap). Domains (Bulbar, Fine Motor, Gross Motor, Respiratory) and their respective ALSFRS-R items are shown vertically; model comparisons are shown horizontally for both RMSE and r.

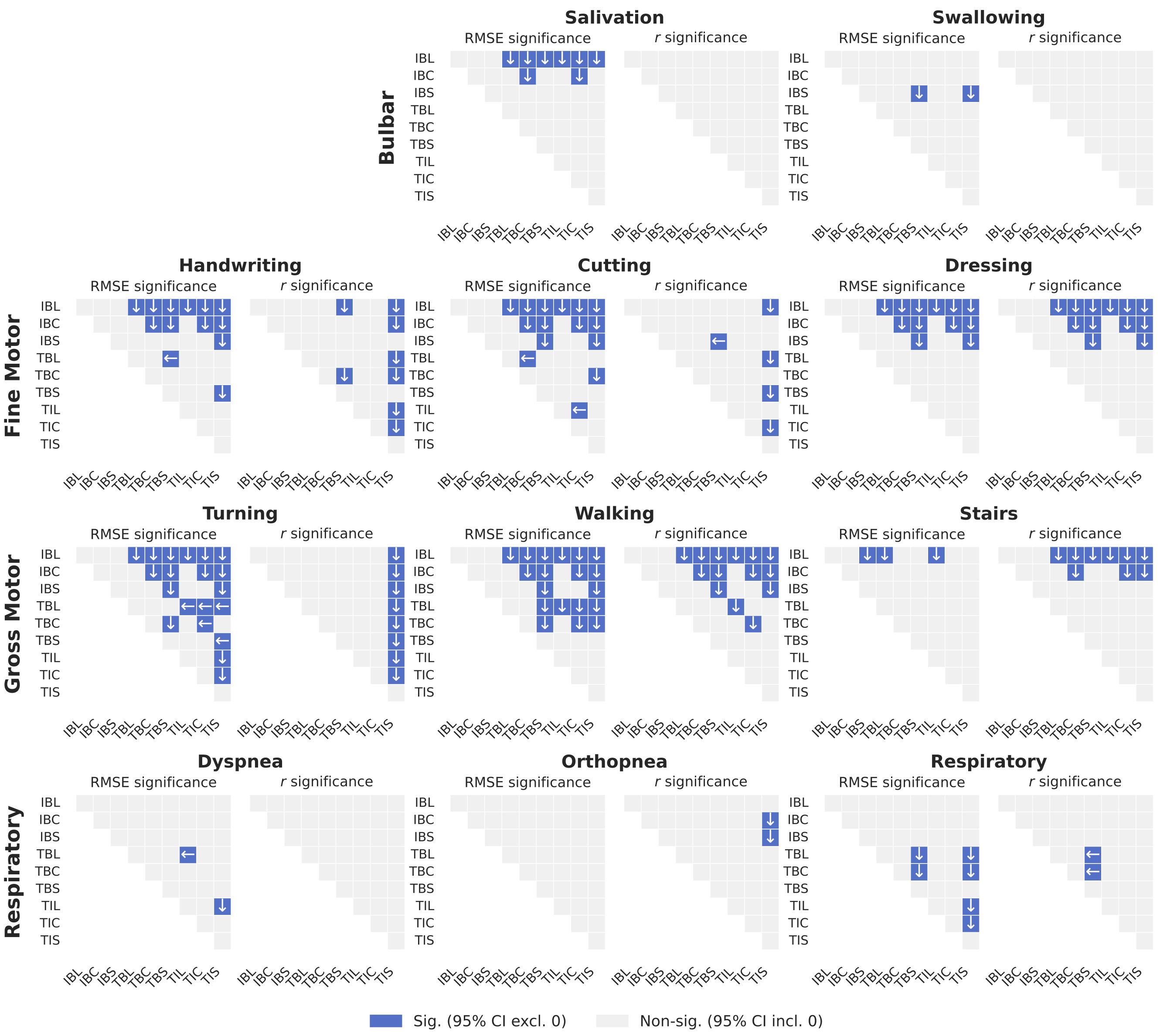

Figure S5. Participant 2 pairwise significance analysis of model performance across ALSFRS-R functional domains and targets. Each cell compares two models (IBL, IBC, IBS, TBL, TBC, TBS, TIL, TIC, TIS) for a specific item, indicating whether the difference in RMSE or r between them is statistically significant (Neutral cells: 95% confidence intervals do not overlap, Color cells: intervals overlap). Domains (Bulbar, Fine Motor, Gross Motor, Respiratory) and their respective ALSFRS-R items are shown vertically; model comparisons are shown horizontally for both RMSE and r.

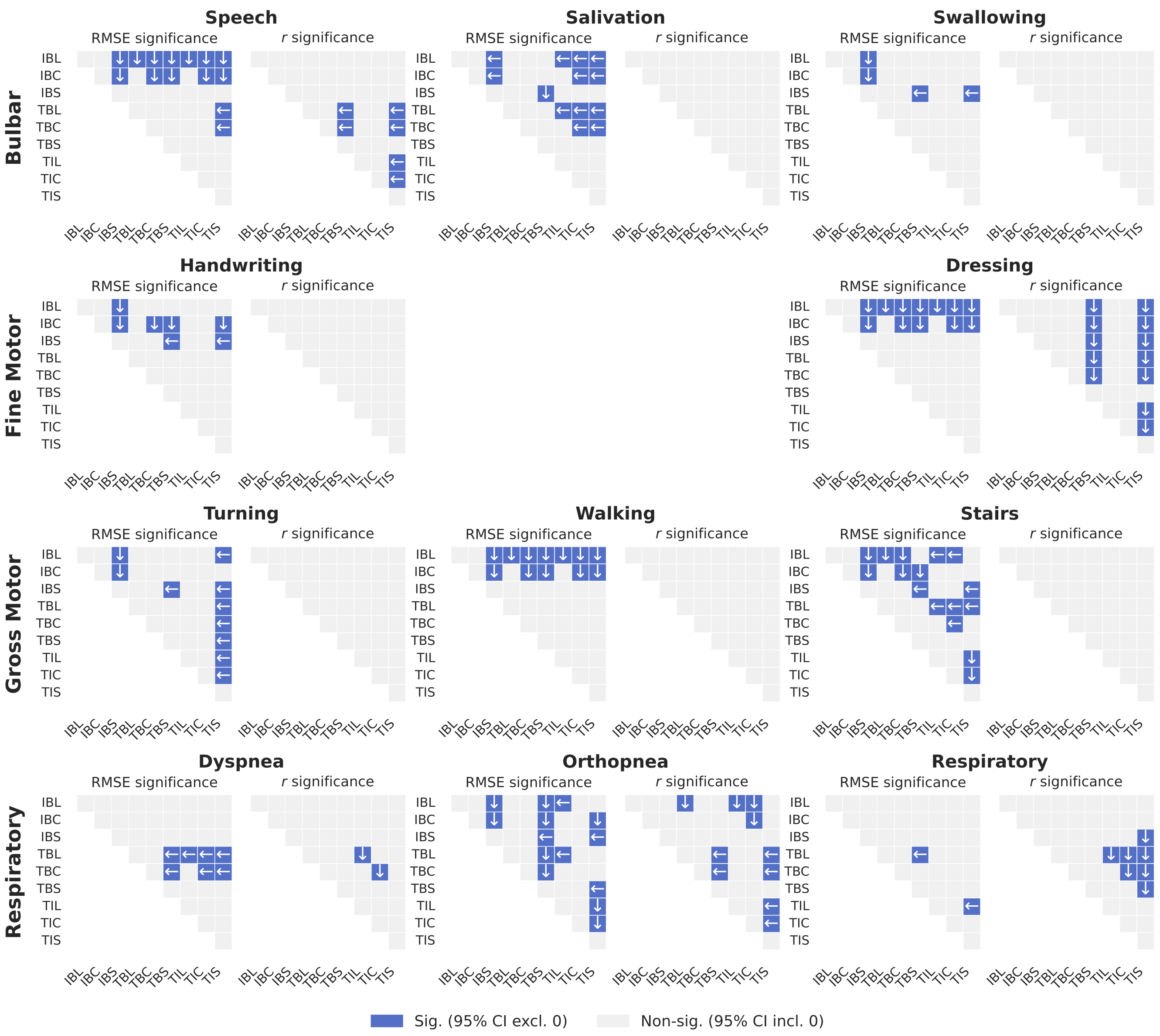

Figure S6. Participant 3 pairwise significance analysis of model performance across ALSFRS-R functional domains and targets. Each cell compares two models (IBL, IBC, IBS, TBL, TBC, TBS, TIL, TIC, TIS) for a specific item, indicating whether the difference in RMSE or r between them is statistically significant (Neutral cells: 95% confidence intervals do not overlap, Color cells: intervals overlap). Domains (Bulbar, Fine Motor, Gross Motor, Respiratory) and their respective ALSFRS-R items are shown vertically; model comparisons are shown horizontally for both RMSE and r.

Table S1. Individual batch mean prediction error (RMSE) by pseudo-labeling interpolation technique and participant ALSFRS-R scale.

Pt.	Domain	ALSFRS-R	Linear Slope	Cubic Polynomial	Self-Attention
			RMSE (95% CI)	RMSE (95% CI)	RMSE (95% CI)
1	Bulbar	Speech	0.28 (0.23 – 0.35)	0.14 (0.09 – 0.19)	0.18 (0.11 – 0.23)
		Salivation	—	—	—
		Swallowing	0.33 (0.29 – 0.36)	0.49 (0.42 – 0.54)	0.19 (0.13 – 0.25)
	Fine Motor	Handwriting	0.54 (0.44 – 0.65)	0.36 (0.31 – 0.39)	0.20 (0.15 – 0.25)
		Cutting	0.18 (0.14 – 0.22)	0.13 (0.11 – 0.15)	0.10 (0.07 – 0.13)
		Dressing	0.33 (0.28 – 0.37)	0.32 (0.27 – 0.37)	0.22 (0.19 – 0.26)
	Gross Motor	Turning	0.32 (0.26 – 0.36)	0.23 (0.16 – 0.28)	0.27 (0.24 – 0.31)
		Walking	0.02 (0.01 – 0.03)	0.02 (0.01 – 0.03)	0.12 (0.10 – 0.14)
		Stairs	0.01 (0.00 – 0.01)	0.00 (0.00 – 0.00)	—
	Respiratory	Dyspnea	—	—	—
		Orthopnea	0.53 (0.41 – 0.62)	0.47 (0.35 – 0.58)	0.45 (0.35 – 0.55)
		Respiratory	—	—	—
	Composite	Composite	1.86 (1.46 – 2.14)	1.69 (1.25 – 2.00)	4.72 (1.09 – 6.88)
2	Bulbar	Speech	—	—	—
		Salivation	0.52 (0.36 – 0.64)	0.51 (0.33 – 0.65)	0.45 (0.30 – 0.58)
		Swallowing	0.17 (0.02 – 0.28)	0.17 (0.05 – 0.27)	0.15 (0.08 – 0.20)
	Fine Motor	Handwriting	0.51 (0.34 – 0.66)	0.50 (0.36 – 0.63)	0.34 (0.16 – 0.49)
		Cutting	0.37 (0.24 – 0.45)	0.43 (0.24 – 0.55)	0.30 (0.13 – 0.44)
		Dressing	0.31 (0.24 – 0.38)	0.38 (0.28 – 0.43)	0.36 (0.25 – 0.40)
	Gross Motor	Turning	0.30 (0.20 – 0.40)	0.30 (0.20 – 0.37)	0.30 (0.18 – 0.41)
		Walking	0.31 (0.25 – 0.35)	0.33 (0.26 – 0.38)	0.26 (0.20 – 0.30)
		Stairs	0.36 (0.28 – 0.42)	0.33 (0.25 – 0.39)	0.21 (0.14 – 0.27)
	Respiratory	Dyspnea	—	—	—
		Orthopnea	0.29 (0.23 – 0.34)	0.31 (0.24 – 0.36)	0.42 (0.32 – 0.50)
		Respiratory	—	—	—
	Composite	Composite	3.01 (2.18 – 3.74)	3.14 (2.37 – 3.78)	4.41 (3.46 – 5.10)
3	Bulbar	Speech	0.45 (0.37 – 0.48)	0.49 (0.41 – 0.51)	0.21 (0.15 – 0.25)
		Salivation	0.10 (0.08 – 0.13)	0.10 (0.06 – 0.14)	0.39 (0.31 – 0.45)
		Swallowing	0.13 (0.09 – 0.16)	0.24 (0.04 – 0.34)	0.00 (0.00 – 0.00)
	Fine Motor	Handwriting	0.16 (0.07 – 0.21)	0.18 (0.12 – 0.23)	0.00 (0.00 – 0.01)
		Cutting	0.02 (0.01 – 0.03)	0.01 (0.00 – 0.01)	—
		Dressing	0.41 (0.33 – 0.46)	0.46 (0.38 – 0.50)	0.18 (0.12 – 0.23)
	Gross Motor	Turning	0.17 (0.09 – 0.25)	0.20 (0.13 – 0.26)	0.07 (0.06 – 0.08)
		Walking	0.43 (0.35 – 0.47)	0.51 (0.42 – 0.54)	0.22 (0.16 – 0.26)
		Stairs	0.12 (0.06 – 0.17)	0.16 (0.08 – 0.22)	0.00 (0.00 – 0.00)
	Respiratory	Dyspnea	—	—	0.19 (0.14 – 0.24)
		Orthopnea	0.13 (0.10 – 0.16)	0.18 (0.14 – 0.21)	0.00 (0.00 – 0.00)
		Respiratory	—	—	0.22 (0.16 – 0.26)
	Composite	Composite	2.27 (1.69 – 2.74)	2.61 (1.98 – 3.08)	4.62 (2.86 – 5.59)

Table S2. Individual batch mean outcome correlation (r) by pseudo-labeling interpolation technique and participant ALSFRS-R scale.

Pt.	Domain	ALSFRS-R	Linear Slope	Cubic Polynomial	Self-Attention
			r (95% CI)	r (95% CI)	r (95% CI)
1	Bulbar	Speech	0.00 (-0.21 – 0.21)	0.00 (-0.21 – 0.21)	–0.15 (-0.37 – 0.09)
		Salivation	—	—	—
		Swallowing	–0.22 (-0.42 – -0.01)	–0.11 (-0.31 – 0.11)	0.18 (-0.05 – 0.40)
	Fine Motor	Handwriting	0.00 (-0.21 – 0.21)	0.00 (-0.21 – 0.21)	–0.20 (-0.41 – 0.03)
		Cutting	0.00 (-0.21 – 0.21)	0.00 (-0.21 – 0.21)	0.00 (-0.23 – 0.23)
		Dressing	0.33 (0.13 – 0.51)	0.58 (0.41 – 0.70)	0.68 (0.54 – 0.79)
	Gross Motor	Turning	–0.09 (-0.30 – 0.12)	–0.41 (-0.57 – -0.21)	0.28 (0.06 – 0.48)
		Walking	0.00 (-0.21 – 0.21)	0.00 (-0.21 – 0.21)	0.31 (0.08 – 0.51)
		Stairs	0.00 (-0.21 – 0.21)	0.00 (-0.21 – 0.21)	—
	Respiratory	Dyspnea	—	—	—
		Orthopnea	0.39 (0.19 – 0.55)	0.31 (0.11 – 0.49)	0.20 (-0.03 – 0.42)
		Respiratory	—	—	—
	Composite	Composite	0.10 (-0.11 – 0.31)	–0.07 (-0.28 – 0.14)	0.06 (-0.17 – 0.29)
2	Bulbar	Speech	—	—	—
		Salivation	0.05 (-0.30 – 0.38)	–0.13 (-0.45 – 0.22)	0.36 (0.01 – 0.62)
		Swallowing	0.00 (-0.34 – 0.34)	0.00 (-0.34 – 0.34)	0.00 (-0.34 – 0.34)
	Fine Motor	Handwriting	–0.01 (-0.35 – 0.33)	0.25 (-0.10 – 0.55)	0.56 (0.27 – 0.76)
		Cutting	–0.13 (-0.45 – 0.22)	0.05 (-0.30 – 0.39)	0.52 (0.21 – 0.73)
		Dressing	0.46 (0.13 – 0.69)	0.13 (-0.22 – 0.45)	0.03 (-0.31 – 0.37)
	Gross Motor	Turning	0.11 (-0.24 – 0.44)	0.07 (-0.28 – 0.41)	0.45 (0.13 – 0.69)
		Walking	0.31 (-0.04 – 0.59)	0.23 (-0.12 – 0.53)	0.05 (-0.30 – 0.39)
		Stairs	0.33 (-0.02 – 0.60)	0.40 (0.07 – 0.66)	0.65 (0.40 – 0.81)
	Respiratory	Dyspnea	—	—	—
		Orthopnea	0.62 (0.35 – 0.79)	0.50 (0.20 – 0.72)	0.53 (0.22 – 0.74)
		Respiratory	—	—	—
	Composite	Composite	0.34 (0.00 – 0.61)	0.43 (0.11 – 0.68)	0.66 (0.41 – 0.82)
3	Bulbar	Speech	–0.13 (-0.45 – 0.23)	–0.23 (-0.53 – 0.12)	–0.04 (-0.39 – 0.31)
		Salivation	–0.22 (-0.53 – 0.13)	–0.16 (-0.47 – 0.20)	0.22 (-0.14 – 0.52)
		Swallowing	0.00 (-0.34 – 0.34)	0.00 (-0.34 – 0.34)	0.00 (-0.35 – 0.35)
	Fine Motor	Handwriting	0.00 (-0.34 – 0.34)	0.00 (-0.34 – 0.34)	0.00 (-0.35 – 0.35)
		Cutting	0.00 (-0.34 – 0.34)	0.00 (-0.34 – 0.34)	—
		Dressing	–0.29 (-0.58 – 0.05)	–0.35 (-0.62 – 0.00)	0.03 (-0.33 – 0.37)
	Gross Motor	Turning	0.00 (-0.34 – 0.34)	0.00 (-0.34 – 0.34)	–0.25 (-0.55 – 0.11)
		Walking	–0.22 (-0.52 – 0.14)	–0.08 (-0.41 – 0.27)	–0.25 (-0.55 – 0.11)
		Stairs	0.00 (-0.34 – 0.34)	0.00 (-0.34 – 0.34)	0.00 (-0.35 – 0.35)
	Respiratory	Dyspnea	—	—	–0.30 (-0.59 – 0.05)
		Orthopnea	0.58 (0.30 – 0.77)	0.59 (0.30 – 0.77)	0.00 (-0.35 – 0.35)
		Respiratory	—	—	–0.31 (-0.59 – 0.04)
	Composite	Composite	–0.29 (-0.57 – 0.06)	–0.20 (-0.51 – 0.15)	–0.04 (-0.38 – 0.31)

Table S3. Transfer batch mean prediction error (RMSE) by pseudo-labeling interpolation technique and participant ALSFRS-R scale.

Pt.	Domain	ALSFRS-R	Linear Slope	Cubic Polynomial	Self-Attention
			RMSE (95% CI)	RMSE (95% CI)	RMSE (95% CI)
1	Bulbar	Speech	0.08 (0.07 – 0.09)	0.07 (0.06 – 0.08)	0.10 (0.08 – 0.10)
		Salivation	—	—	—
		Swallowing	0.24 (0.22 – 0.25)	0.29 (0.27 – 0.31)	0.11 (0.08 – 0.15)
	Fine Motor	Handwriting	0.45 (0.40 – 0.48)	0.47 (0.42 – 0.51)	0.12 (0.09 – 0.14)
		Cutting	0.12 (0.10 – 0.13)	0.07 (0.06 – 0.08)	0.07 (0.05 – 0.08)
		Dressing	0.29 (0.26 – 0.31)	0.21 (0.17 – 0.24)	0.31 (0.26 – 0.34)
	Gross Motor	Turning	0.35 (0.30 – 0.39)	0.32 (0.25 – 0.36)	0.29 (0.24 – 0.32)
		Walking	0.29 (0.24 – 0.32)	0.29 (0.25 – 0.32)	0.16 (0.12 – 0.20)
		Stairs	0.04 (0.04 – 0.05)	0.04 (0.03 – 0.04)	—
	Respiratory	Dyspnea	0.54 (0.48 – 0.59)	0.54 (0.48 – 0.60)	0.41 (0.25 – 0.55)
		Orthopnea	0.14 (0.10 – 0.16)	0.11 (0.08 – 0.14)	0.12 (0.10 – 0.14)
		Respiratory	—	—	—
	Composite	Composite	3.50 (2.53 – 4.33)	2.15 (1.58 – 2.87)	4.21 (3.25 – 4.94)
2	Bulbar	Speech	0.30 (0.25 – 0.34)	0.31 (0.25 – 0.34)	0.20 (0.14 – 0.25)
		Salivation	0.18 (0.15 – 0.21)	0.17 (0.13 – 0.20)	0.27 (0.15 – 0.33)
		Swallowing	0.05 (0.03 – 0.06)	0.04 (0.03 – 0.05)	0.04 (0.03 – 0.05)
	Fine Motor	Handwriting	0.08 (0.04 – 0.11)	0.12 (0.08 – 0.13)	0.15 (0.13 – 0.16)
		Cutting	0.06 (0.04 – 0.07)	0.11 (0.08 – 0.12)	0.07 (0.05 – 0.09)
		Dressing	0.11 (0.04 – 0.17)	0.10 (0.04 – 0.15)	0.12 (0.06 – 0.16)
	Gross Motor	Turning	0.03 (0.02 – 0.04)	0.05 (0.04 – 0.06)	0.02 (0.02 – 0.02)
		Walking	0.15 (0.11 – 0.18)	0.19 (0.14 – 0.22)	0.09 (0.07 – 0.10)
		Stairs	0.24 (0.19 – 0.27)	0.25 (0.20 – 0.28)	0.23 (0.17 – 0.30)
	Respiratory	Dyspnea	0.48 (0.39 – 0.54)	0.51 (0.43 – 0.56)	0.38 (0.27 – 0.48)
		Orthopnea	0.30 (0.24 – 0.34)	0.31 (0.26 – 0.35)	0.33 (0.28 – 0.35)
		Respiratory	0.34 (0.27 – 0.38)	0.40 (0.32 – 0.45)	0.19 (0.13 – 0.24)
	Composite	Composite	4.97 (3.36 – 6.22)	4.85 (3.25 – 6.20)	8.28 (5.46 – 9.71)
3	Bulbar	Speech	0.14 (0.12 – 0.15)	0.13 (0.11 – 0.14)	0.19 (0.14 – 0.23)
		Salivation	0.14 (0.10 – 0.18)	0.11 (0.07 – 0.14)	0.20 (0.12 – 0.24)
		Swallowing	0.09 (0.06 – 0.12)	0.10 (0.06 – 0.13)	0.10 (0.07 – 0.12)
	Fine Motor	Handwriting	0.05 (0.02 – 0.08)	0.06 (0.03 – 0.08)	0.06 (0.04 – 0.07)
		Cutting	0.02 (0.01 – 0.03)	0.01 (0.01 – 0.02)	—
		Dressing	0.18 (0.14 – 0.19)	0.18 (0.14 – 0.21)	0.13 (0.10 – 0.16)
	Gross Motor	Turning	0.15 (0.13 – 0.17)	0.15 (0.13 – 0.17)	0.18 (0.16 – 0.19)
		Walking	0.15 (0.12 – 0.17)	0.17 (0.14 – 0.19)	0.20 (0.15 – 0.24)
		Stairs	0.02 (0.01 – 0.03)	0.03 (0.02 – 0.04)	0.05 (0.03 – 0.06)
	Respiratory	Dyspnea	0.10 (0.07 – 0.12)	0.11 (0.08 – 0.13)	0.21 (0.15 – 0.24)
		Orthopnea	0.14 (0.12 – 0.16)	0.16 (0.12 – 0.19)	0.05 (0.04 – 0.06)
		Respiratory	0.11 (0.09 – 0.12)	0.14 (0.11 – 0.16)	0.22 (0.16 – 0.25)
	Composite	Composite	2.60 (1.86 – 3.12)	2.77 (2.01 – 3.26)	3.67 (2.19 – 5.10)

Table S4. Transfer batch mean outcome correlation (r) by pseudo-labeling interpolation technique and participant ALSFRS-R scale.

Pt.	Domain	ALSFRS-R	Linear Slope	Cubic Polynomial	Self-Attention
			r (95% CI)	r (95% CI)	r (95% CI)
1	Bulbar	Speech	0.00 (-0.20 – 0.20)	0.00 (-0.20 – 0.20)	−0.05 (-0.27 – 0.19)
		Salivation	—	—	—
		Swallowing	0.84 (0.77 – 0.89)	0.82 (0.75 – 0.88)	0.75 (0.63 – 0.84)
	Fine Motor	Handwriting	0.00 (-0.20 – 0.20)	0.00 (-0.20 – 0.20)	−0.05 (-0.28 – 0.18)
		Cutting	0.00 (-0.20 – 0.20)	0.00 (-0.20 – 0.20)	0.00 (-0.23 – 0.23)
		Dressing	0.49 (0.33 – 0.63)	0.85 (0.78 – 0.90)	−0.13 (-0.35 – 0.11)
	Gross Motor	Turning	−0.11 (-0.30 – 0.09)	−0.02 (-0.22 – 0.18)	0.05 (-0.18 – 0.28)
		Walking	0.00 (-0.20 – 0.20)	0.00 (-0.20 – 0.20)	0.73 (0.60 – 0.82)
		Stairs	0.00 (-0.20 – 0.20)	0.00 (-0.20 – 0.20)	—
	Respiratory	Dyspnea	0.51 (0.35 – 0.64)	0.39 (0.21 – 0.54)	−0.20 (-0.41 – 0.03)
		Orthopnea	0.98 (0.96 – 0.98)	0.97 (0.96 – 0.98)	0.99 (0.98 – 0.99)
		Respiratory	—	—	—
	Composite	Composite	−0.36 (-0.52 – -0.17)	−0.49 (-0.62 – -0.32)	0.74 (0.61 – 0.83)
2	Bulbar	Speech	0.46 (0.13 – 0.69)	0.50 (0.19 – 0.72)	0.72 (0.49 – 0.85)
		Salivation	−0.31 (-0.59 – 0.04)	−0.40 (-0.65 – -0.07)	−0.13 (-0.45 – 0.23)
		Swallowing	0.00 (-0.34 – 0.34)	0.00 (-0.34 – 0.34)	0.00 (-0.34 – 0.34)
	Fine Motor	Handwriting	0.35 (0.00 – 0.62)	0.00 (-0.34 – 0.34)	0.62 (0.35 – 0.79)
		Cutting	−0.20 (-0.51 – 0.15)	0.01 (-0.33 – 0.35)	−0.48 (-0.71 – -0.16)
		Dressing	0.95 (0.91 – 0.98)	0.96 (0.92 – 0.98)	0.92 (0.85 – 0.96)
	Gross Motor	Turning	0.41 (0.08 – 0.66)	0.45 (0.13 – 0.69)	−0.14 (-0.46 – 0.22)
		Walking	0.95 (0.89 – 0.97)	0.87 (0.75 – 0.93)	0.96 (0.92 – 0.98)
		Stairs	0.92 (0.85 – 0.96)	0.93 (0.86 – 0.96)	0.79 (0.62 – 0.89)
	Respiratory	Dyspnea	0.89 (0.78 – 0.94)	0.84 (0.70 – 0.92)	0.90 (0.80 – 0.95)
		Orthopnea	0.56 (0.26 – 0.76)	0.59 (0.31 – 0.78)	0.85 (0.71 – 0.92)
		Respiratory	0.61 (0.34 – 0.79)	0.48 (0.16 – 0.71)	−0.26 (-0.55 – 0.10)
	Composite	Composite	0.21 (-0.14 – 0.52)	0.23 (-0.12 – 0.53)	−0.12 (-0.45 – 0.23)
3	Bulbar	Speech	0.25 (-0.06 – 0.52)	0.31 (0.00 – 0.57)	−0.57 (-0.77 – -0.28)
		Salivation	0.00 (-0.31 – 0.31)	0.00 (-0.31 – 0.31)	−0.01 (-0.35 – 0.34)
		Swallowing	0.00 (-0.31 – 0.31)	0.00 (-0.31 – 0.31)	0.00 (-0.35 – 0.35)
	Fine Motor	Handwriting	0.00 (-0.31 – 0.31)	0.00 (-0.31 – 0.31)	0.00 (-0.35 – 0.35)
		Cutting	0.00 (-0.31 – 0.31)	0.00 (-0.31 – 0.31)	—
		Dressing	0.02 (-0.29 – 0.33)	0.16 (-0.16 – 0.45)	0.75 (0.54 – 0.87)
	Gross Motor	Turning	0.00 (-0.31 – 0.31)	0.00 (-0.31 – 0.31)	0.24 (-0.12 – 0.54)
		Walking	−0.33 (-0.58 – -0.02)	−0.37 (-0.61 – -0.07)	−0.43 (-0.68 – -0.09)
		Stairs	0.00 (-0.31 – 0.31)	0.00 (-0.31 – 0.31)	0.00 (-0.35 – 0.35)
	Respiratory	Dyspnea	0.07 (-0.25 – 0.37)	−0.08 (-0.39 – 0.23)	−0.09 (-0.42 – 0.27)
		Orthopnea	0.94 (0.89 – 0.97)	0.87 (0.77 – 0.93)	0.00 (-0.35 – 0.35)
		Respiratory	−0.28 (-0.55 – 0.03)	−0.20 (-0.48 – 0.12)	−0.32 (-0.60 – 0.03)
	Composite	Composite	−0.58 (-0.76 – -0.33)	−0.54 (-0.73 – -0.27)	−0.50 (-0.72 – -0.18)

Table S5. Transfer incremental mean prediction error (RMSE) by pseudo-labeling interpolation technique and participant ALSFRS-R scale.

Pt.	Domain	ALSFRS-R	Linear Slope	Cubic Polynomial	Self-Attention
			RMSE (95% CI)	RMSE (95% CI)	RMSE (95% CI)
1	Bulbar	Speech	0.05 (0.04 – 0.05)	0.05 (0.04 – 0.05)	0.03 (0.03 – 0.04)
		Salivation	—	—	—
		Swallowing	0.24 (0.22 – 0.25)	0.28 (0.26 – 0.30)	0.17 (0.10 – 0.24)
	Fine Motor	Handwriting	0.17 (0.15 – 0.19)	0.17 (0.15 – 0.19)	0.11 (0.09 – 0.13)
		Cutting	0.16 (0.12 – 0.19)	0.16 (0.12 – 0.19)	0.03 (0.03 – 0.04)
		Dressing	0.34 (0.28 – 0.38)	0.42 (0.37 – 0.45)	0.28 (0.23 – 0.32)
	Gross Motor	Turning	0.27 (0.21 – 0.32)	0.24 (0.16 – 0.29)	0.28 (0.24 – 0.32)
		Walking	0.32 (0.27 – 0.36)	0.32 (0.26 – 0.36)	0.15 (0.11 – 0.19)
		Stairs	0.08 (0.07 – 0.09)	0.08 (0.07 – 0.09)	—
	Respiratory	Dyspnea	0.49 (0.44 – 0.53)	0.47 (0.43 – 0.50)	0.30 (0.25 – 0.34)
		Orthopnea	0.11 (0.09 – 0.12)	0.11 (0.09 – 0.13)	0.13 (0.09 – 0.16)
		Respiratory	—	—	—
	Composite	Composite	3.02 (2.74 – 3.27)	3.31 (3.04 – 3.51)	3.39 (2.22 – 4.70)
2	Bulbar	Speech	0.29 (0.24 – 0.32)	0.29 (0.24 – 0.33)	0.20 (0.14 – 0.25)
		Salivation	0.16 (0.12 – 0.20)	0.16 (0.12 – 0.20)	0.28 (0.17 – 0.33)
		Swallowing	0.04 (0.03 – 0.05)	0.04 (0.03 – 0.05)	0.05 (0.03 – 0.06)
	Fine Motor	Handwriting	0.09 (0.06 – 0.13)	0.12 (0.07 – 0.15)	0.10 (0.08 – 0.12)
		Cutting	0.03 (0.02 – 0.04)	0.07 (0.05 – 0.08)	0.05 (0.04 – 0.06)
		Dressing	0.05 (0.04 – 0.06)	0.04 (0.03 – 0.05)	0.08 (0.05 – 0.11)
	Gross Motor	Turning	0.13 (0.11 – 0.14)	0.14 (0.12 – 0.16)	0.08 (0.06 – 0.09)
		Walking	0.05 (0.04 – 0.06)	0.07 (0.05 – 0.08)	0.07 (0.05 – 0.08)
		Stairs	0.24 (0.20 – 0.27)	0.24 (0.20 – 0.28)	0.23 (0.17 – 0.28)
	Respiratory	Dyspnea	0.63 (0.57 – 0.67)	0.54 (0.41 – 0.60)	0.44 (0.32 – 0.53)
		Orthopnea	0.29 (0.25 – 0.33)	0.31 (0.26 – 0.34)	0.33 (0.29 – 0.36)
		Respiratory	0.35 (0.27 – 0.40)	0.40 (0.32 – 0.46)	0.19 (0.16 – 0.21)
	Composite	Composite	4.66 (3.31 – 5.90)	4.66 (3.22 – 5.89)	3.92 (2.62 – 5.09)
3	Bulbar	Speech	0.17 (0.14 – 0.19)	0.18 (0.14 – 0.20)	0.21 (0.17 – 0.23)
		Salivation	0.36 (0.26 – 0.41)	0.36 (0.26 – 0.41)	0.31 (0.22 – 0.35)
		Swallowing	0.15 (0.09 – 0.19)	0.15 (0.09 – 0.19)	0.17 (0.12 – 0.21)
	Fine Motor	Handwriting	0.11 (0.07 – 0.14)	0.11 (0.07 – 0.14)	0.08 (0.05 – 0.11)
		Cutting	0.03 (0.01 – 0.04)	0.03 (0.01 – 0.04)	—
		Dressing	0.16 (0.11 – 0.18)	0.16 (0.12 – 0.19)	0.12 (0.09 – 0.14)
	Gross Motor	Turning	0.19 (0.16 – 0.20)	0.18 (0.16 – 0.20)	0.29 (0.26 – 0.30)
		Walking	0.14 (0.11 – 0.16)	0.14 (0.12 – 0.16)	0.20 (0.15 – 0.23)
		Stairs	0.26 (0.21 – 0.28)	0.26 (0.22 – 0.28)	0.06 (0.04 – 0.08)
	Respiratory	Dyspnea	0.38 (0.28 – 0.44)	0.39 (0.28 – 0.46)	0.32 (0.24 – 0.37)
		Orthopnea	0.32 (0.22 – 0.38)	0.24 (0.16 – 0.30)	0.11 (0.08 – 0.12)
		Respiratory	0.09 (0.08 – 0.10)	0.10 (0.08 – 0.12)	0.16 (0.11 – 0.21)
	Composite	Composite	2.27 (1.43 – 2.95)	2.21 (1.34 – 2.83)	4.44 (2.91 – 5.46)

Table S6. Transfer incremental mean outcome correlation (r) by pseudo-labeling interpolation technique and participant ALSFRS-R scale.

Pt.	Domain	ALSFRS-R	Linear Slope	Cubic Polynomial	Self-Attention
			r (95% CI)	r (95% CI)	r (95% CI)
1	Bulbar	Speech	0.00 (-0.20 – 0.20)	0.00 (-0.20 – 0.20)	0.66 (0.51 – 0.77)
		Salivation	—	—	—
		Swallowing	0.86 (0.80 – 0.90)	0.86 (0.80 – 0.90)	0.15 (-0.08 – 0.37)
	Fine Motor	Handwriting	0.00 (-0.20 – 0.20)	0.00 (-0.20 – 0.20)	0.25 (0.02 – 0.46)
		Cutting	0.00 (-0.20 – 0.20)	0.00 (-0.20 – 0.20)	0.00 (-0.23 – 0.23)
		Dressing	−0.26 (-0.43 – -0.06)	−0.06 (-0.26 – 0.14)	−0.29 (-0.49 – -0.06)
	Gross Motor	Turning	0.16 (-0.04 – 0.34)	0.12 (-0.08 – 0.31)	−0.01 (-0.24 – 0.23)
		Walking	0.00 (-0.20 – 0.20)	0.00 (-0.20 – 0.20)	0.73 (0.60 – 0.82)
		Stairs	0.00 (-0.20 – 0.20)	0.00 (-0.20 – 0.20)	—
	Respiratory	Dyspnea	0.85 (0.79 – 0.90)	0.79 (0.70 – 0.85)	0.60 (0.42 – 0.73)
		Orthopnea	0.99 (0.99 – 0.99)	0.99 (0.98 – 0.99)	0.99 (0.98 – 0.99)
		Respiratory	—	—	—
	Composite	Composite	0.51 (0.35 – 0.64)	0.50 (0.34 – 0.64)	0.71 (0.57 – 0.81)
2	Bulbar	Speech	0.72 (0.50 – 0.85)	0.75 (0.55 – 0.87)	0.78 (0.59 – 0.89)
		Salivation	−0.14 (-0.46 – 0.21)	−0.35 (-0.62 – -0.01)	−0.10 (-0.42 – 0.26)
		Swallowing	0.00 (-0.34 – 0.34)	0.00 (-0.34 – 0.34)	0.00 (-0.34 – 0.34)
	Fine Motor	Handwriting	0.39 (0.05 – 0.64)	0.14 (-0.21 – 0.46)	0.84 (0.71 – 0.92)
		Cutting	0.30 (-0.05 – 0.58)	−0.07 (-0.40 – 0.28)	0.60 (0.32 – 0.78)
		Dressing	0.99 (0.98 – 0.99)	0.99 (0.98 – 1.00)	0.98 (0.95 – 0.99)
	Gross Motor	Turning	0.57 (0.28 – 0.76)	0.52 (0.21 – 0.73)	0.91 (0.83 – 0.96)
		Walking	0.99 (0.98 – 0.99)	0.99 (0.97 – 0.99)	0.96 (0.92 – 0.98)
		Stairs	0.93 (0.87 – 0.97)	0.94 (0.88 – 0.97)	0.89 (0.79 – 0.95)
	Respiratory	Dyspnea	0.95 (0.89 – 0.97)	0.95 (0.90 – 0.98)	0.92 (0.85 – 0.96)
		Orthopnea	0.67 (0.42 – 0.82)	0.66 (0.42 – 0.82)	0.87 (0.75 – 0.93)
		Respiratory	0.64 (0.38 – 0.80)	0.48 (0.16 – 0.70)	0.31 (-0.03 – 0.59)
	Composite	Composite	0.61 (0.34 – 0.79)	0.61 (0.34 – 0.79)	0.68 (0.44 – 0.83)
3	Bulbar	Speech	0.26 (-0.06 – 0.53)	0.26 (-0.06 – 0.53)	−0.41 (-0.66 – -0.07)
		Salivation	0.00 (-0.31 – 0.31)	0.00 (-0.31 – 0.31)	0.08 (-0.28 – 0.41)
		Swallowing	0.00 (-0.31 – 0.31)	0.00 (-0.31 – 0.31)	0.00 (-0.35 – 0.35)
	Fine Motor	Handwriting	0.00 (-0.31 – 0.31)	0.00 (-0.31 – 0.31)	0.00 (-0.35 – 0.35)
		Cutting	0.00 (-0.31 – 0.31)	0.00 (-0.31 – 0.31)	—
		Dressing	0.09 (-0.23 – 0.39)	0.23 (-0.09 – 0.50)	0.79 (0.61 – 0.89)
	Gross Motor	Turning	0.00 (-0.31 – 0.31)	0.00 (-0.31 – 0.31)	0.32 (-0.04 – 0.60)
		Walking	−0.32 (-0.57 – -0.01)	−0.37 (-0.61 – -0.07)	−0.43 (-0.67 – -0.09)
		Stairs	0.00 (-0.31 – 0.31)	0.00 (-0.31 – 0.31)	0.00 (-0.35 – 0.35)
	Respiratory	Dyspnea	0.63 (0.40 – 0.79)	0.59 (0.34 – 0.76)	0.18 (-0.18 – 0.50)
		Orthopnea	0.95 (0.91 – 0.97)	0.96 (0.93 – 0.98)	0.00 (-0.35 – 0.35)
		Respiratory	0.76 (0.59 – 0.87)	0.77 (0.60 – 0.87)	0.83 (0.67 – 0.91)
	Composite	Composite	−0.69 (-0.82 – -0.48)	−0.63 (-0.78 – -0.39)	0.08 (-0.28 – 0.42)
